# Carbon-based quantum dots enhance platelets aggregation through migrasomes biogenesis

**DOI:** 10.1186/s12951-025-04010-9

**Published:** 2026-01-17

**Authors:** Ang Li, Leiliang Zhang

**Affiliations:** 1 Department of Clinical Laboratory Medicine, The First Affiliated Hospital of Shandong First Medical University & Shandong Provincial Qianfoshan Hospital, Jinan, Shandong China; 2 School of Pharmaceutical Sciences & Institute of Materia Medica, Shandong First Medical University & Shandong Academy of Medical Sciences, Jinan, Shandong China; 3 Department of Pathogen Biology, School of Clinical and Basic Medical Sciences, Shandong First Medical University & Shandong Academy of Medical Sciences, Jinan, Shandong China

**Keywords:** CQD, GQD, GOQDs, Migrasomes, Platelet aggregation

## Abstract

While previous research has primarily focused on nanoparticles larger than 20 nm for promoting migrasome formation, this study shifts the emphasis to nanoparticles smaller than 10 nm. Our findings reveal the significant impact of these sub-10 nm carbon-based quantum dots, specifically carbon quantum dots (CQD), graphene quantum dots (GQD), and graphitic oxide quantum dots (GOQDs), on migrasome formation, mitochondrial integrity, and platelet function. We establish a clear correlation between the application of these nanoparticles and the induction of migrasomes. Furthermore, our results indicate that CQD, GQD, and GOQDs enhance migrasome formation by upregulating phosphatidylinositol 4,5-bisphosphate (PI(4,5)P2), cholesterol, and GTP-RhoA. Additionally, these nanoparticles provide mitochondrial protection through a process called mitocytosis and promote increased platelet aggregation. This study represents a pioneering effort to connect various types of nanoscale graphene materials with the dynamic behavior of migrasomes, thereby advancing our understanding of nanoscale materials and their biological implications.

**Graphical Abstract:**

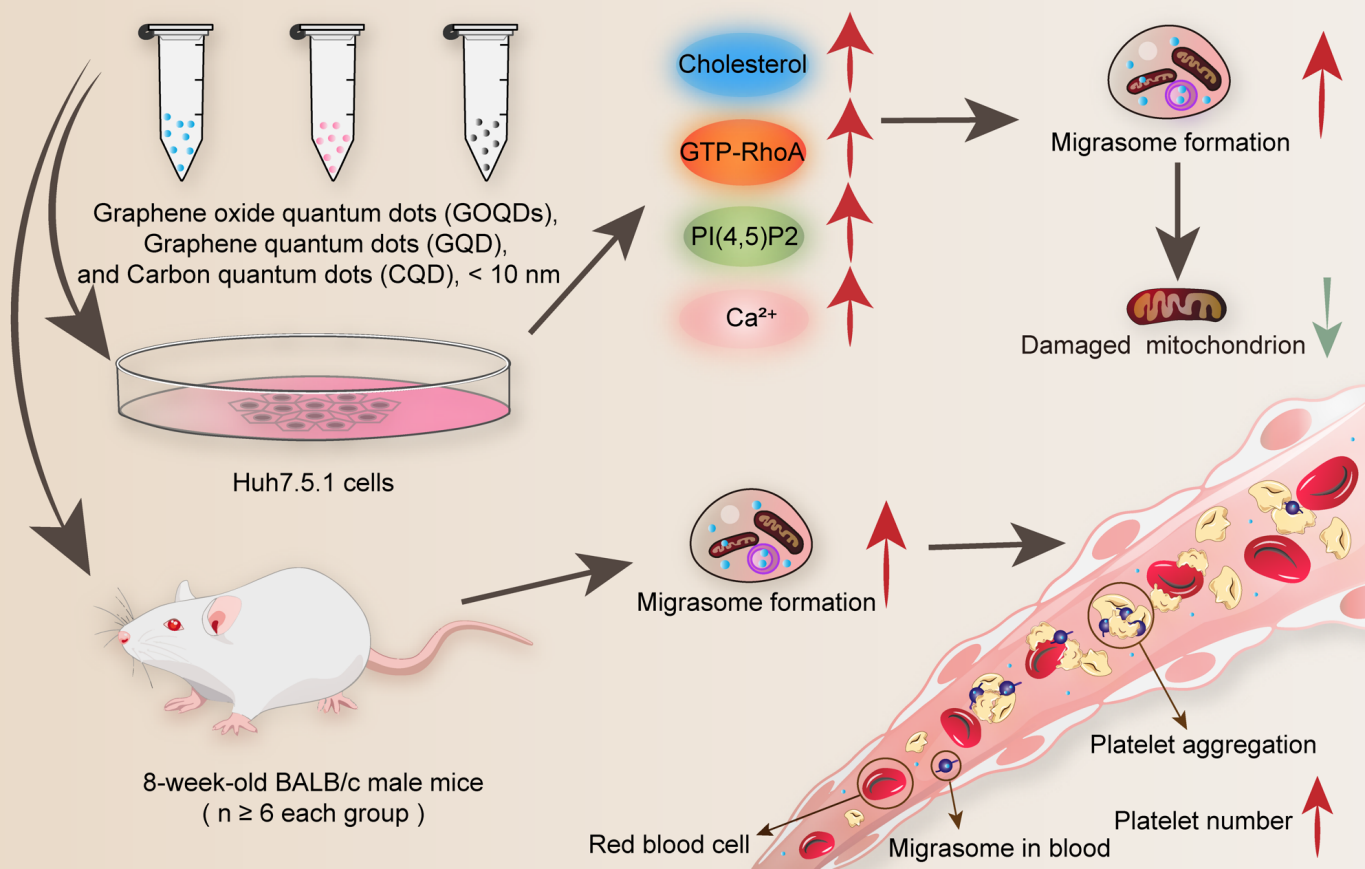

**Supplementary Information:**

The online version contains supplementary material available at 10.1186/s12951-025-04010-9.

## Introduction

Carbon quantum dots (CQD), graphene quantum dots (GQD), and graphene oxide quantum dots (GOQDs) represent a family of nanomaterials that exhibit unique optical and electronic properties due to their nanoscale size and carbon-based structure [1–3]. These nanoparticles, typically having a diameter of less than 10 nm, have garnered significant interest in biomedical applications due to their stable carbon composition, nanoscale dimensions, exceptional photochemical properties, superior biocompatibility, large specific surface area, strong tissue penetration capabilities, and efficient biological labeling potential [4, 5]. Naturally occurring carbon quantum dots are ubiquitous in the environment and are produced during various processes, such as caramel production [6], coffee bean roasting [7], and pastry baking [8]. Additionally, metal-doped CQD have exhibited effects on bacteria, plants, and mouse C2C12 cells [9]. Despite the growing interest in their applications, investigations into their effects on cell biology remain relatively limited. Recent studies have also indicated that CQD influence platelet activity and that newly developed composite materials based on CQD can promote wound healing [6, 10]. These findings prompt us to explore whether CQD affect migrasomes, which are integral components of the coagulation system and play a critical role in wound healing.

Migrasomes, which are associated with retraction fibers formed by actin, are increasingly recognized as organelles [11]. A key player in this process is RhoA, which regulates cytoskeletal remodeling [12]. The activation of RhoA stimulates Rho-associated protein kinase 1 (ROCK1), which further contributes to migrasome formation [13]. Additionally, cholesterol is required for migrasome formation [14]. Phosphatidylinositol-4,5-bisphosphate (PI(4,5)P2), activated by PIP5K1A [15], plays an essential role in migrasome formation [16]. Together, RhoA, ROCK1, cholesterol, and PI(4,5)P2 are integral to understanding the mechanisms that underlie migrasome biology.

Previous studies have shown that polystyrene and zinc oxide nanoparticles with diameters ranging from 28 to 100 nm can enter migrasomes [17, 18], and it has been observed that the diameter of nanomaterials is inversely proportional to the quantity of migrasomes formed. However, there are currently no reports investigating the effects of materials with diameters smaller than 10 nm on migrasomes. This gap in research motivates our selection of carbon quantum dot, a material that is abundant in nature, highly biocompatible, low in cytotoxicity, and widely applicable. Given their excellent cell adaptability and the broad scope of material preparation research, the structural diversity of CQD makes them an ideal candidate for studying the effects of extremely small nanomaterials on cellular migrasomes.

In this study, we investigated the concentration-dependent effects of these nanoparticles on migrasome formation, along with their interactions with cellular signaling pathways. Moreover, our study included in vivo experiments utilizing mouse models, which provided additional insights into the biological implications of QDs treatment in a physiological context. This study aims to elucidate the role of CQD, GQD, and GOQDs in regulating migrasome formation, mitochondrial protection, and platelet function, thereby highlighting their potential as multifunctional nanotherapeutics.

## Materials and methods

### Materials and solution Preparation

CQD (104963, 102636), GQD (102017), 5 nm gold nanoparticles in water solution (101609), GOQDs (106104) and GOQDs in water solution (100082) were obtained from XFNANO (Nanjing, China). A stock solution of CQD at a concentration of 1 mg/mL was prepared in phosphate-buffered saline (PBS), and the GQD stock solution at a concentration of 5 mg/mL was prepared in 10% dimethyl sulfoxide (DMSO). The CQD and GQD were vortexed for 1 min (VORTEX-5, Kylin-Bell, Haimen, China) and sonicated for 20 min at 40 Hz (SCIENTZ, Ningbo, China), then stored in suspension at 4 °C. Prior to cell treatment, the materials were re-dispersed by vortex mixing for 5 min and diluted to various concentrations using culture medium.

### Optical characterization

Photoluminescence and photoluminescence excitation spectra were measured using a HITACHI F-7000 spectrophotometer. The emission spectrum of CQD was recorded from 580 to 700 nm upon excitation at 565 nm, while the excitation spectrum was collected by setting the emission wavelength to 610 nm and scanning the excitation from 400 to 600 nm. The emission spectrum of GQD was recorded from 560 to 570 nm upon excitation at 800 nm, and the excitation spectrum was obtained by setting the emission wavelength to 610 nm and scanning the excitation from 350 to 600 nm. For GOQDs, the emission spectrum was recorded from 400 to 600 nm upon excitation at 350 nm, with the excitation spectrum collected by setting the emission wavelength to 450 nm and scanning the excitation from 270 to 430 nm. Both excitation and emission slit widths were set to 5.0 nm. The samples were dissolved in toluene at a concentration of 0.1 mg/mL, and the solution was degassed by nitrogen bubbling for 10 min prior to measurement.

### Fourier transform infrared (FT-IR) spectroscopy

Powder samples were prepared using the conventional KBr pellet method and analyzed with a FT-IR spectrometer (INVENIO S, Bruker, Billerica, MA, USA) [19].

### DLS, PDI and zeta potential

Stock solutions were diluted in deionized water or in cell culture media (DMEM + 10% FBS). The dynamic light scattering (DLS) [20], polydispersity index (PDI) [21], and zeta potential [21] of each QD solution were measured using a Zetasizer Pro (Malvern Panalytical, UK).

### Plasmids and reagents

The GFP-tagged PH domain of phospholipase C delta 1 (PH-PLCD1-GFP) (Addgene_51407) was obtained from Addgene (USA) and was a gift from Dr. Tamas Balla [22]. The mCherry-tagged 2x-Rhotekin-GBD (Addgene_214142) was obtained from Addgene and were generously provided by Dr. Reza Ahmadian at Heinrich Heine University in Düsseldorf, Germany [23]. The mCherry-tagged TSPAN4 (mCherry-TSPAN4) was provided by Dr. Li Yu from Tsinghua University, China [24]. Cells were cultured in appropriate cell culture medium, and plasmid transfection was performed using FuGENE HD (E2311, Promega, Madison, WI, USA) according to the manufacturer’s guidelines. ISA2011B (HY-16937), NSC23766 (HY-15723), carbonyl cyanide 3-chlorophenylhydrazone (CCCP) (HY-100941), and filipin complex (HY-N6716) were acquired from MedChemExpress (Princeton, NJ, USA). FITC-labeled WGA (GTX01502) was obtained from GeneTex (Irvine, CA, USA), while TRITC-labeled phalloidin (CA1610) and FITC-labeled phalloidin (CA1620) were sourced from Solarbio (Beijing, China). Dasabuvir (T3489) was purchased from Target Molecule Corp (Boston, MA, USA).

### Cell culture and transfection

The Huh7.5.1 cell line is a liver cancer cell line that was kindly provided by Dr. Francis Chisari of the Scripps Research Institute in La Jolla, CA. The cells were cultured in high-glucose Dulbecco’s modified Eagle’s medium (DMEM) (SH30022.01, HyClone, Marlborough, MA, USA), supplemented with 10% fetal bovine serum (FBS) (FSP500, ExCell Bio, Shanghai, China), 100 U/mL penicillin, and 100 µg/mL streptomycin (SV30010, HyClone, Marlborough, MA, USA), and incubated at 37 °C in a 5% CO₂ atmosphere. Cells were passaged every two to three days using 0.25% Trypsin-EDTA (25200-056, Gibco, Thermo Fisher Scientific, Waltham, MA, USA) and were routinely tested for mycoplasma contamination. For cell transfection, 70% confluent cultured cells in a 2 cm dish were transfected with 1 µg of DNA using 2.5 µL of FuGENE HD.

### Cell counting kit-8 (CCK-8) assay

Huh7.5.1 cells were seeded at approximately 1 × 10^4^ cells per well in 96-well plates and incubated for 24 h. After being treated with 0–110 µg/mL CQD, GQD and GOQDs for 17 h, the cells were incubated with 10 µL of CCK-8 reagent (RRID: AB_2314126, Proteintech, Wuhan, China) for an additional 1 h. The absorbance was measured at 450 nm using a Multiskan GO microplate reader (Thermo Fisher, USA). Dots-only blanks were excluded to avoid optical interference. The experiment was divided into several treatment groups, and each group was repeated at least six times to ensure accuracy.

### Scratch wound cell migration assay

Huh7.5.1 cells were cultured to confluence in 6-well plates containing DMEM with 10% FBS. A scratch was made across the center of the well using a P1000 pipette tip, and the medium was immediately replaced. Images were captured using inverted microscope (Novel Optics, Nanjing, China) at 24 h post-scratch. Brightfield images of the same region of interest (ROI) were taken to visualize the leading edge. The migration distance of the cell edge at each time point was measured using ImageJ software.

### Immunofluorescence analysis

For migrasome staining, the cells seeded on glass coverslips (YA0350, Solarbio, Beijing, China) were fixed with 4% paraformaldehyde (P1110, Solarbio, Beijing, China), and stained with either 3 µg/mL WGA, TRITC-labeled phalloidin (A1620, Solarbio, Beijing, China), or 40 µM filipin for 30 min.

Confocal images were acquired using a Thermo Fisher Scientific EVOS M7000 digital confocal microscopeor an Olympus FV12-IXCOV confocal microscope (Japan) at a resolution of 1024 × 1024 pixels. Additionally, fluorescence images were captured using an ECHO RVL2-K microscope (USA).

### High resolution transmission electron microscopy (HRTEM)

CQD, GQD and GOQDs were diluted in 100% alcohol, and then dropped on copper mesh to dry. Images were captured using a HRTEM (Talos F200i, RRID: SCR_019911, thermo scientific, USA).

### Transmission electron microscopy (TEM)

Cells from each experimental group were collected and washed twice with PBS. After fixation with 2.5% glutaraldehyde EM fixative at 4 °C 17 h, the samples were sent to the SCICLUBS platform (Hangzhou, Zhejiang, China) for TEM analysis (H-7800, HITACHI, Japan).

### Measurement of mitochondrial membrane potential

The Huh7.5.1 cells were treated with culture medium containing either CQD or no CQD, along with 15 µM dasabuvir (T3489, Target Molecule Corp, Boston, MA, USA) and 4 µM CCCP for a duration of 17 h. After this treatment, the cells were incubated with the MT-1 working solution for 30 min, in accordance with the instructions provided by the MT-1 MitoMP Detection Kit (MT13, DOJINDO, Japan).

### Reactive oxygen species (ROS) detection

Huh7.5.1 cells were treated with CQD, GQD, and GOQDs for 17 h, followed by washing with PBS. The cells were then incubated in working solution of DCFH-DA from the ROS detection kit (S0034S, Beyotime, Shanghai, China) at 37 °C for 20 min. After rinsing the cells with PBS, they were resuspended in phenol-free red DMEM. The DCF fluorescence intensity was measured at an excitation wavelength of 488 nm and an emission wavelength of 525 nm using a microplate reader (SpectraMax M2, Molecular Devices, California, USA).

### Superoxide dismutase (SOD) activity assay

Huh7.5.1 cells were treated with CQD, GQD, and GOQDs for 17 h, then washed three times with cold PBS at 4 °C. The cells were scraped in ice-cold PBS and homogenized using a glass homogenizer. The slurry was centrifuged at 12,000 × g for 15 min at 4 °C, and the supernatant was collected. For the total SOD activity assay (NBT method) (S0109, Beyotime, Shanghai, China), 20 µL of the supernatant per sample was added to 160 µL of NBT/enzyme solution, followed by the addition of 20 µL of reaction starter working fluid. The mixture was incubated at 37 °C for 30 min. The absorbance was measured at 560 nm using a microplate reader (SpectraMax M2, Molecular Devices, California, USA).

### Cytoplasmic calcium ion detection

Huh7.5.1 cells treated with CQD, GQD, and GOQD underwent calcium ion staining after 17 h. Cytoplasmic calcium ion levels were assessed using the Fluo-4/AM probe (YEASEN, 40704ES50) at a concentration of 4 µM, visualized in green. The probe was prepared with 0.04% Pluronic F-127 (YEASEN, 60318ES60) and 0.5 mM Probenecid to enhance solubility and cellular retention. After a one-hour incubation for staining, the cells were thoroughly rinsed with PBS to remove excess probe. The samples were then resuspended in PBS for observation using a fluorescence microscope.

### Detection of active RhoA by GST-pull down assay

RhoA activity was detected using pull-down assays with GST-Rhotekin-RBD. Huh7.5.1 cell lysates were incubated with 100 µl of glutathione resin conjugated with GST-Rhotekin-RBD, which were synthetic plasmids sourced from GENEWIZ (NJ, USA) and expressed in BL21(DE3), for 1.5 h at 4 °C to isolate GTP-bound RhoA and Rac1. The complexes were then centrifuged at 500 g for 6 min and washed three times with wash buffer. Following the washes, the eluted proteins were resuspended in loading buffer (P0015, Beyotime, Shanghai, China) for SDS-PAGE and subsequent Western blot analysis.

Samples were probed for specific proteins with the following primary antibodies: rabbit anti-RhoA (10749-1-AP, Proteintech, Wuhan, China) and mouse anti-GAPDH (60004-1-Ig, Proteintech, Wuhan, China). After thorough washing, the membranes were incubated with HRP-conjugated secondary Goat Anti-Rabbit antibody (AB_856214, Proteintech, Wuhan, China) or HRP-conjugated secondary Goat Anti-Mouseantibody (AB_10890902, Proteintech, Wuhan, China). Bands were visualized using chemiluminescence with the Clarity Western BeyoECL substrate (P0018FM, Beyotime, Shanghai, China). Band images were captured using the ChemiDoc MP imaging system (BIO-RAD, Hercules, CA, USA). Densitometric analysis was conducted with Image J software, and the resulting data were presented in arbitrary optical density (OD) units.

### Animal treatment, blood smear, and platelet counting

The ethical approval for the experimental animals was obtained from Shandong First Medical University (W202504160624). Eight-week-old male BALB/c mice were purchased from Jinan Pengyue Experimental Animal Breeding (Shandong, China). The mice were housed in a specific pathogen-free environment at a temperature of 23 ± 1 °C and 60 ± 10% relative humidity, with a one-week adaptation period prior to treatment. The mice were randomly divided into three groups, with six mice per group. Before treatment, 20 µL of diluted peripheral blood from the tail of each mouse was mixed with 380 µL of platelet dilution (R20341, Yuanye, Shanghai, China) and counted using a hemocytometer. Additionally, 3–5 µL of tail peripheral blood was used to prepare blood smears. The blood smears were fixed in methanol for 10 min and then stained with Wright’s staining solution for another 10 min (C0135, Beyotime, Shanghai, China). Subsequently, the mice received intraperitoneal injections of 3 µg/g body weight CQD, 6 µg/g body weight GQD, and 6 µg/g body weight GOQDs, all diluted in saline. After 24 h, blood was collected from the tail tip, and the sample was diluted for platelet counting. Simultaneously, blood smears were prepared and stained using Wright’s staining solution.

### Extraction of migrasomes from mouse whole blood

Eight-week-old male BALB/c mice were intraperitoneally injected with 3 µg/g body weight CQD, 6 µg/g body weight GQD, 6 µg/g body weight GOQDs, or physiological saline, and then kept for 7 days in a sterile, quiet, and dark environment at 26 °C. After 7 days, whole blood was collected from the mice, and the mouse whole blood migrasomes were extracted using Migralso™ Pro Mouse Immune Cells extraction kit (MGS-P007-MS, Migrasome Therapeutics, Beijing, China). The purification of migrasomes was confirmed by Western blot with antibodies against CPQ (HPA023235-100UL, SIGMA), EGOT (ab190693, Abcam), ITGAV (ab179475, Abcam) and NDST1 (WH0003340M1, SIGMA). The extracted migrasomes were then dissolved in PBS and stained with 2 µg/ml WGA for 30 min. Subsequently, the samples were evenly dispersed on slides pre-treated with BSA for observation and imaging.

### Raman spectrum

Huh7.5.1 cells were treated with QDs for 48 h, then subjected to trypsinization for 90 s and resuspended in phenol-free DMEM. Eight-week-old male BALB/c mice were intraperitoneally injected with 3 µg/g body weight of CQD, 6 µg/g body weight of GQD, and 6 µg/g body weight of GOQDs. The mice were then maintained for 7 days in a sterile, quiet, and dark environment at 26 °C. After 7 days, whole blood was collected from the mice and centrifuged to obtain red blood cell pellets. The pellets were dissolved in an ammonium oxalate solution and then ground using a glass homogenizer. The mixture was centrifuged at 4,000 × g for 15 min to remove red cell debris, followed by a centrifugation at 20,000 × g for 60 min to pellet the QDs. The resulting solutions were prepared on silicon substrates and measured using an Ar laser (102 nm) based Raman spectrometer (QE65Pro, Ocean Insight, USA) [19].

### Tail tip bleeding assay

Mice were anesthetized using isoflurane inhalation and positioned on a flat surface. A 5 mm segment of the tail tip was amputated, and blood was collected within 3 min using a 20 µL capillary blood collection tube (10–20 µL, Laixu, Shandong, China) pre-treated with EDTA (ST063, Beyotime, Shanghai, China). The severed tail was then immediately immersed in warm PBS (50 µL) supplemented with 40 mM EDTA and allowed to bleed for 10 min. The remaining blood mixture was then placed on a clear plastic film for photographic documentation.

### Statistical analysis

Statistical analysis of fluorescence intensity and Western blot signals was performed using ImageJ software, while statistical significance was assessed with GraphPad Prism 8 (GraphPad Software, Inc., La Jolla, CA).Statistical significance for platelet counts was calculated using a paired sample t-test, while a two-tailed unpaired t-test was used for other experiments. All data are presented as means ± SEM. NS denotes *p* > 0.05; *, *p* < 0.05; **, *p* < 0.01; ***, *p* < 0.001; ****, *p* < 0.0001.

## Results

### Characterization and cytotoxicity assessment of GQD, GOQDs and CQD

**Fig. 1:**
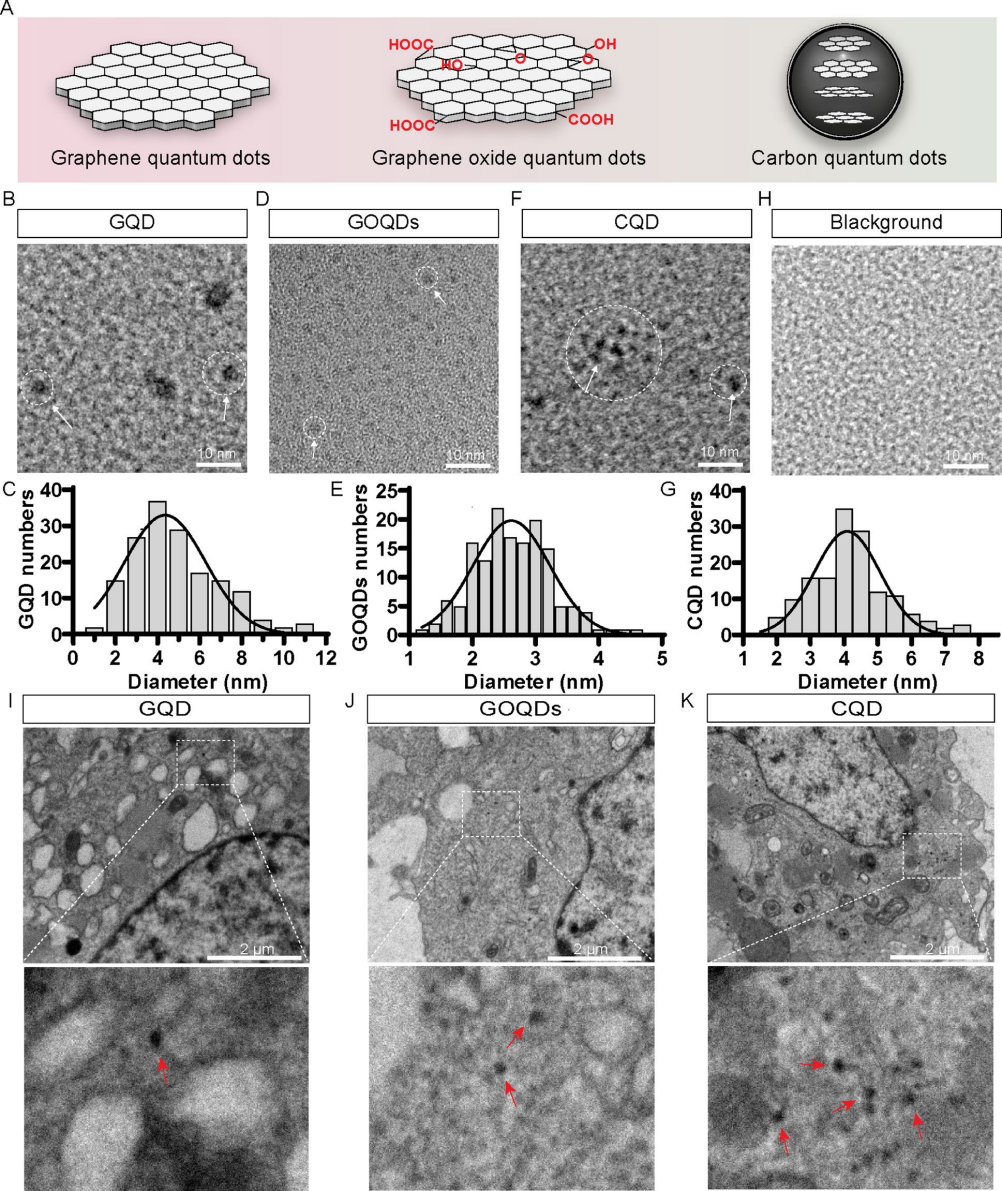
Morphological characterization and internalization of GQD, GOQDs and CQD. (**A**) A schematic representation illustrating the structural differences between graphene quantum dots (GQD), graphene oxide quantum dots (GOQDs), and carbon quantum dots (CQD). (**B**) High resolution transmission electron microscopy (HRTEM) images of GQD. Scale bar: 10 nm. (**C**) Histogram showing the particle size distribution of GQD derived from HRTEM analysis, with over 163 particles evaluated. Average diameter: 4.363 ± 1.927 nm. (**D**) HRTEM images of GOQDs. Scale bar: 10 nm. (**E**) Histogram of the particle size distribution of GOQDs obtained from HRTEM images, with more than 151 particles analyzed. Average diameter: 2.617 ± 0.6097 nm. (**F**) HRTEM images of CQD. Scale bar: 10 nm. (**G**) Histogram of the particle size distribution of CQD from HRTEM images, with over 149 particles analyzed. Average diameter: 4.091 ± 0.9755 nm. (**H**) HRTEM images of the blank control are presented to illustrate the background. Scale bar: 10 nm. (**I**) TEM images of Huh7.5.1 cells internalized with 20 µg/mL GQD for 17 h. Particles are indicated by red arrows. Scale bars: 2 μm. (**J**) TEM images of Huh7.5.1 cells internalized with 20 µg/mL GOQDs for 17 h. Particles are indicated by red arrows. Scale bars: 2 μm. (**K**) TEM images of Huh7.5.1 cells after 17 h of internalization with 10 µg/mL CQD. Particles are indicated by red arrows. Scale bars: 2 μm

The CQD family encompasses GQD, GOQDs, CQD and others, all of which are carbon-based structures with diameters less than 10 nm. Schematic representations of GQD, GOQDs, and CQD are illustrated in Fig. 1A. Using HRTEM, we observed that GQD were predominantly disc-shaped, with an average size of 4.363 ± 1.927 nm (Fig. 1B and C). GOQDs appear in evenly dispersed discs, with an average size of 2.617 ± 0.6097 nm (Fig. 1D and E). CQD were predominantly irregular spherical, with an average size of 4.091 ± 0.9755 nm (Fig. 1F and G). Figure 1H is the blank control of HRTEM image.

Next, TEM was employed to assess the internalization of GQD, GOQDs, and CQD in Huh7.5.1 cells. TEM images demonstrated clear internalization of GQD at a concentration of 20 µg/mL after 17 h, with particles distinctly visible within the cells, indicated by red arrows (Fig. 1I). Similarly, GOQDs at 20 µg/mL for 17 h showed successful internalization, with well-defined particles confirmed by red arrows (Fig. 1J). CQD was internalized at a lower concentration of 10 µg/mL over the same duration, with their presence also marked by red arrows in the TEM images (Fig. 1K). Overall, TEM analysis confirms that GQD, GOQDs, and CQD can be effectively internalized in Huh7.5.1 cells, highlighting their potential for cellular imaging and drug delivery applications.

**Fig. 2:**
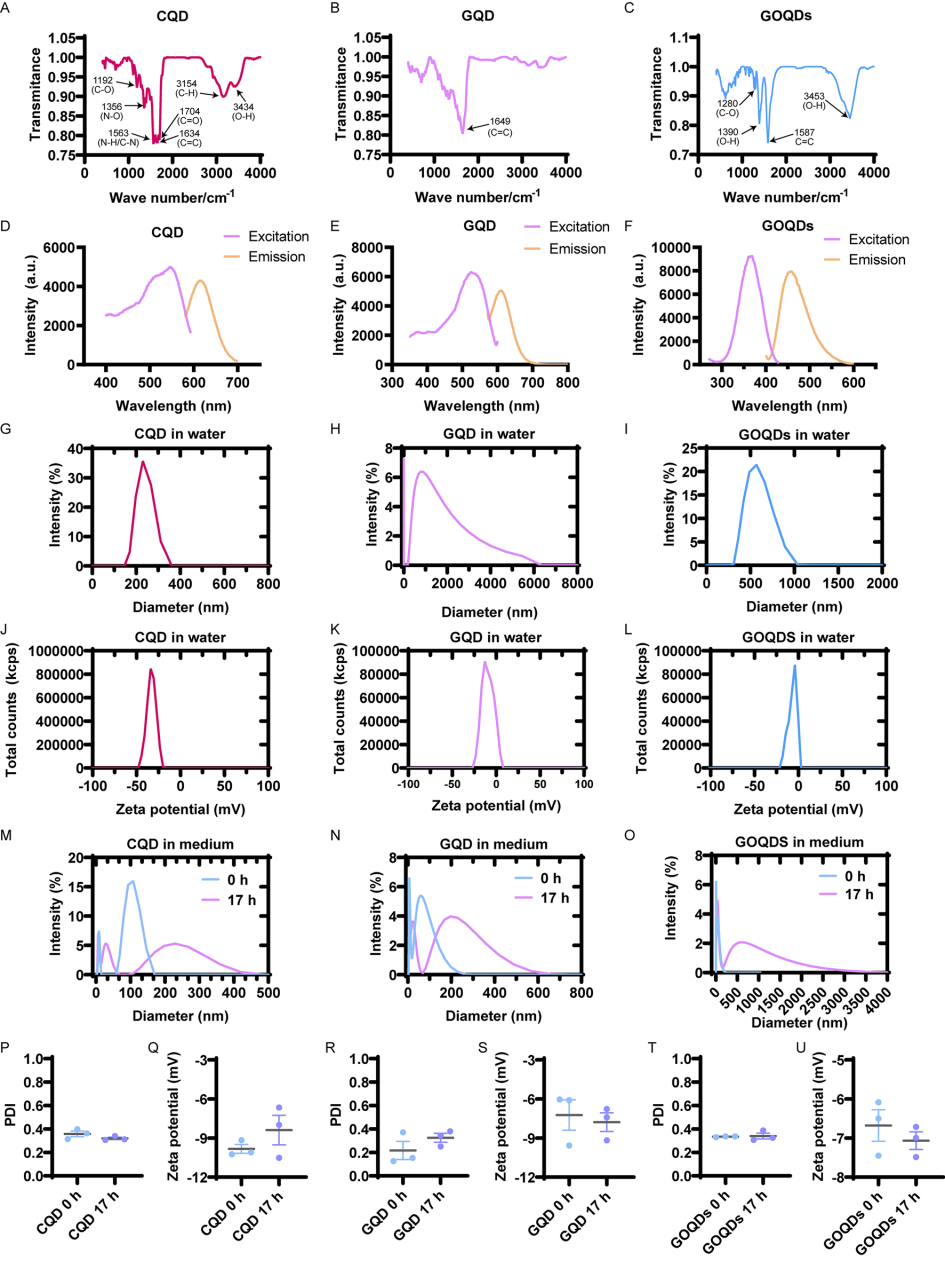
Characterization of GQD, GOQDs and CQD. (**A**) FT-IR spectrum of CQD. (**B**) FT-IR spectrum of GQD. (**C**) FT-IR spectrum of GOQDs. (**D**) Excitation–emission map of CQD. (**E**) Excitation–emission map of GQD. (**F**) Excitation–emission map of GOQDs. (**G**) DLS of CQD in water. 236.4 ± 66.5 nm. (**H**) DLS of GQD in water. 1251 ± 1081 nm. (**I**) DLS of GOQDs in water. 571 ± 261 nm. (**J**) Zeta potential of CQD. (**K**) Zeta potential of GQD. (**L**) Zeta potential of GOQDs. (**M**) DLS of CQD in medium at 0–17 h. (**N**) DLS of GQD in medium at 0–17 h. (**O**) DLS of GOQDs in medium at 0–17 h. (**P**) PDI of CQD in medium at 0–17 h. (**Q**) Zeta potential of CQD in medium at 0–17 h. (**R**) PDI of GQD in medium at 0–17 h. (**S**) Zeta potential of GQD in medium at 0–17 h. (**T**) PDI of GOQDs in medium for 0–17 h. (**U**) Zeta potential of GOQDs in medium at 0–17 h

To analyze the chemical structure and functionality of GQD, GOQDs, and CQD, we conducted FT-IR experiments. FT-IR spectrum of CQD showed O-H bond, C-H bond, C = O bond, C = C bond, N-H/C-N bond, N-O bond and C-O bond at 3170 cm^− 1^, 3154 cm^− 1^, 1704 cm^− 1^, 1634 cm^− 1^, 1563 cm^− 1^, 1356 cm^− 1^ and 1192 cm^− 1^, respectively (Fig. 2A). This indicates that CQD possesses a variety of functional group modifications and complex surface chemistries. FT-IR spectrum of GQD showed only C = C bond at 1649 cm^− 1^, indicating the pure carbon hydrophobic structure of GQD (Fig. 2B). FT-IR spectrum of GOQDs revealed showing O-H bond telescopic vibration, C = C bond, O-H bond in-plane bending vibration, and C-O bond at 3453 cm^− 1^, 1587 cm^− 1^, 1390 cm^− 1^, and 1280 cm^− 1^, respectively (Fig. 2C). This suggests that GOQDs are highly oxidized and contain oxygen-rich functional groups, such as hydroxyl and carboxyl groups.

By determining the excitation spectra and emission spectra of CQD, GQD, and GOQDS, the optical characteristics of these three QDs were clarified, and the spontaneous fluorescence of these materials was confirmed (Fig. 2D, E and F). QDs tend to form larger particles in aqueous solutions (Fig. 2G − I). In contrast, when they are dispersed in a medium, the particle aggregation is less noticeable (Fig. 2M − O). Specifically, the aggregation of materials in culture media is smaller than in aqueous solutions, as shown below. CQD has a size of 236.4 nm in water (Fig. 2G), whereas in the medium, its size is 75.4 nm at 0 h and increases to 106.5 nm by 17 h (Fig. 2M). GQD measures 572 nm in water (Fig. 2H), but in the medium, its size is 38.6 nm at 0 h and 46.17 nm at 17 h (Fig. 2N). Similarly, GOQDs are 571.5 nm in water (Fig. 2I), while in the medium, they show sizes of 11.6 nm at 0 h and 68.5 nm at 17 h (Fig. 2O). Over time, the particles in the medium tend to increase in size. Although GQD is non-water-soluble material that forms large aggregates in aqueous solutions (Fig. 2H), they disperse better and form smaller particles in serum-containing culture media (Fig. 2N). The zeta potential and polydispersity index (PDI) of the three materials in the medium exhibited minimal changes (Fig. 2P − U) and were lower than the values observed in aqueous solutions (Fig. 2J − L). Specifically, the zeta potential results indicate that the dispersion stability of all three materials is superior in aqueous solutions compared to culture media. For instance, CQD in water shows a zeta potential of -33.05 mV (Fig. 2J), while in the medium, the zeta potential is -9.835 mV at 0 h and − 8.39 mV at 17 h (Fig. 2Q). Similarly, GQD has a zeta potential of -10.64 mV in water (Fig. 2K), but in the medium, it measures − 7.233 mV at 0 h and − 7.783 mV at 17 h (Fig. 2S). GOQDs exhibit a zeta potential of -8.18 mV in water (Fig. 2L), whereas in the medium, their values are − 6.679 mV at 0 h and − 7.066 mV at 17 h (Fig. 2U).

To evaluate the cell viability of GQD in Huh7.5.1 cells, we administered various concentrations of GQD for 17 h and conducted CCK-8 assays. Concentrations exceeding 90 µg/mL induced cytotoxicity in Huh7.5.1 cells (Fig. S1A). Next, we treated the cells with 20 µg/mL GQD for varying durations, with subsequent CCK-8 assays indicating that this concentration did not elicit cytotoxic effects over a 48-h period (Fig. S1B). Continuing our assessment, we examined the cell viability of GOQDs in Huh7.5.1 cells. After administering various concentrations for 17 h and performing CCK-8 assays, we observed that doses exceeding 100 µg/mL resulted in cytotoxicity (Fig. S1C). Further, when cells were treated with 40 µg/mL GOQDs for different time intervals, the subsequent CCK-8 assays revealed no cytotoxicity over 48 h (Fig. S1D). In addition, we explored the effects of CQD on Huh7.5.1 cells. Administration of various concentrations for 17 h indicated that concentrations below 110 µg/mL did not induce cytotoxicity (Fig. S1E). Moreover, exposure to 30 µg/mL CQD over different durations yielded results from repeated CCK-8 assays showing that this concentration did not lead to toxicity over a 48-h exposure (Fig. S1F).

### CQD induces migrasomes formation

To assess the potential of CQD in promoting cell migration, we performed a cell scratch assay using Huh7.5.1 cells treated with CQD at concentrations ranging from 0 to 10 µg/mL. The results revealed that CQD concentrations of 3–10 µg/mL significantly enhanced cell migration compared to the untreated control (Fig. S2A and S2B). The migration of Huh7.5.1 cells was assessed following treatment with a concentration of 20 µg/mL CQD over a time course of 0 to 72 h. A significant increase in migration distance was observed at 24, 48, and 72 h post-treatment compared to the mock treatment (Fig. S2C and S2D). These findings indicate that treatment with 20 µg/mL CQD enhances the migratory capacity of Huh7.5.1 cells over time, suggesting a potential role for CQD in modulating cell migration dynamics.

**Fig. 3:**
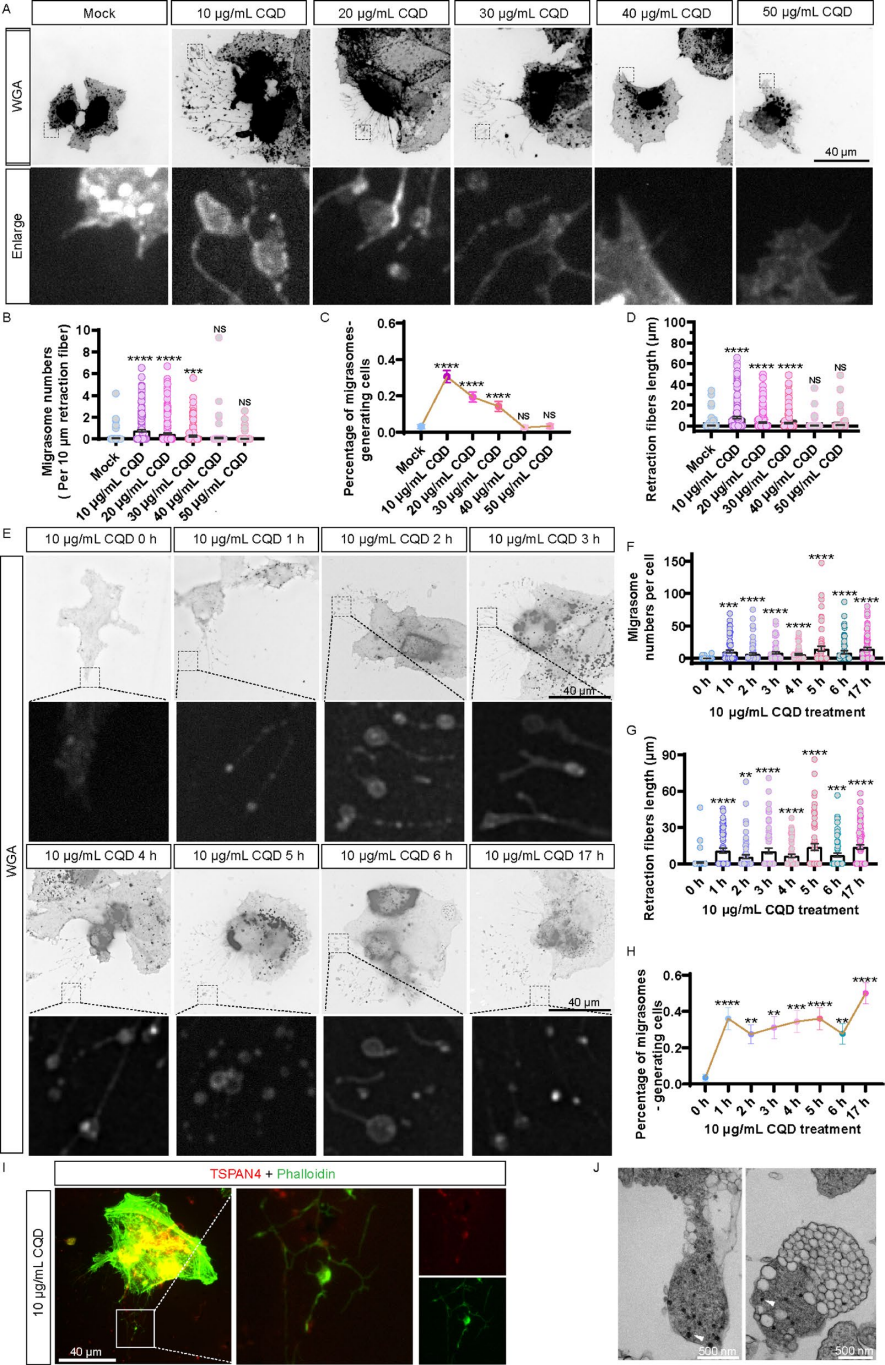
CQD promotes migrasomes formation. (**A**) Huh7.5.1 cells treated with various concentrations of CQD for 5 h. Scale bar: 40 μm. (**B**–**D**) Statistical analyses of migrasome counts, the percentage of migrasome-generating cells, and retraction fiber lengths in panel A. (**E**) Huh7.5.1 cells treated with CQD. Scale bar: 40 μm. (**F **– **H**) Statistical analyses of migrasome numbers per cell, retraction fiber lengths, and the percentage of migrasome-generating cells in panelE. (**I**) Huh7.5.1 cells treated with CQD for 17 h. Scale bar: 40 μm. (**J**) TEM images displaying the migrasome formation process in the left panel and free migrasomes in the right panel of Huh7.5.1 cells treated with 10 µg/mL CQD for 17 h. White triangle indicates CQD located within a migrasome. Scale bar: 500 nm. NS denotes *p* > 0.05; *, *p* < 0.05; **, *p* < 0.01; ***, *p* < 0.001; ****, *p* < 0.0001

To further investigate the induction of migrasomes by CQD, we treated Huh7.5.1 cells with 10 µg/mL CQD for 48 h and conducted staining with WGA, a dye known for labeling migrasomes. Images captured during this process revealed the formation of migrasomes (Fig. S3). We also examined the effects of varying concentrations of CQD on migrasome formation over a 5-h treatment period. CQD with concentrations of 10–30 µg/mL effectively induced migrasome formation (Fig. 3A − D). To confirm the induction of migrasomes by CQD, we treated Huh7.5.1 cells with 10 µg/mL CQD for different durations and stained them with WGA. Images captured from 0 to 17 h post-treatment illustrated the progressive formation of migrasomes (Fig. 3E − H). Additionally, CQD treatment induced the formation of retraction fibers in Huh7.5.1 cells, as evidenced by phalloidin staining (Fig. 3I). Moreover, we employed TEM to further investigate the cellular structures. After a 5-h treatment with 10 µg/mL CQD, we identified pomegranate-like structures that closely resemble previously characterized migrasomes [24]. Notably, we observed CQD encapsulated within these migrasomes (Fig. 3J). Morevoer, we conducted experiments using 5 nm gold nanomaterials as a control to investigate their effects on migrasome formation in Huh7.5.1 cells. Our findings showed that gold nanomaterials do not promote migrasome generation (Fig. S4A and S4B). This result suggests that the effect observed with CQD is indeed specific to carbon-based materials rather than a general phenomenon associated with ultrasmall nanoparticles.

### Levels of cholesterol, GTP-RhoA and PI(4,5)P2 are upregulated by CQD

**Fig. 4:**
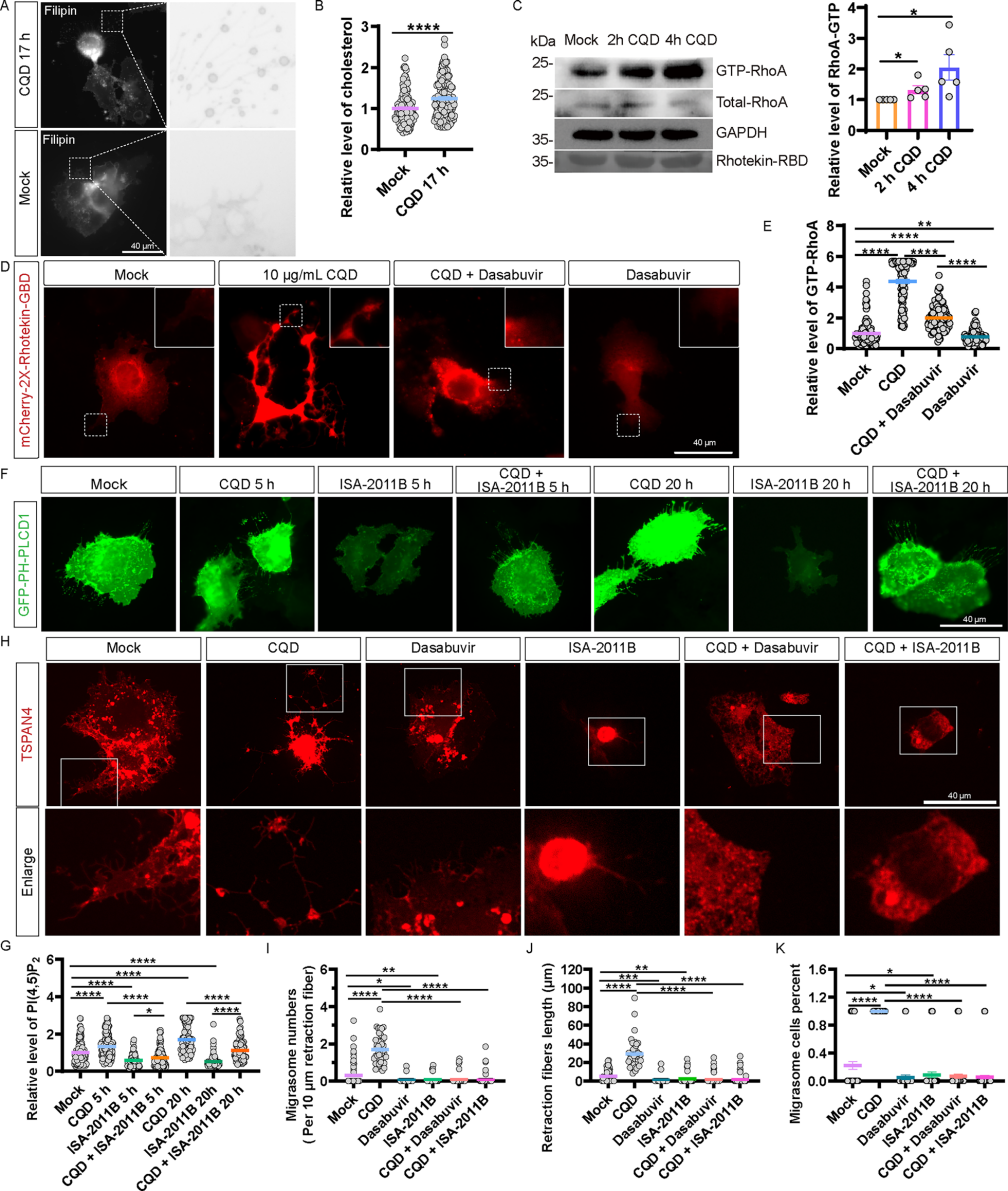
CQD upregulates cholesterol, RhoA activity, and PI(4,5)P2. (**A**) Huh7.5.1 cells were stained with filipin. Scale bar: 40 μm. (**B**) Statistical analysis of panel A. (**C**) GTP-RhoA activation in Huh7.5.1 cells treated with 10 µg/mL CQD. (**D**) Huh7.5.1 cells expressing mCherry-2×-Rhotekin-GBD treated with or without CQD, 15 µM dasabuvir, or a combination of both for 2 h. Scale bar: 40 μm. (**E**) Statistical analysis of panel D. (**F**) Images of Huh7.5.1 cells expressing PH-GFP-PLCD1 treated with or without 10 µg/mL CQD, 100 µM ISA-2011B, or a combination of both. Scale bar: 40 μm. (**G**) Statistical analysis of panel F. (**H**) Converted gray-value mode images of dasabuvir and ISA-2011B effectively inhibit CQD-induced migrasome formation. Scale bar: 40 μm. (**I** – **K**) Statistical analysis of migrasome counts per cell, retraction fiber lengths, and the percentage of migrasome-generating cells from panel H

Cholesterol plays a critical role in the formation of migrasomes [14]. To investigate the effect of CQD on cellular cholesterol levels, Huh7.5.1 cells were treated with 10 µg/mL of CQD for 17 h, followed by filipin staining for visualization. Our results demonstrated a significant increase in cholesterol levels in the CQD-treated cells compared to the control group (Fig. 4A and B).

The RhoA/ROCK1 signaling pathway is known to be pivotal in the biogenesis of migrasomes [13]. To elucidate the relationship between CQD and RhoA, we conducted a GST pull-down assay, confirming a notable increase in GTP-RhoA levels in the CQD-treated group as early as 2 h post-treatment (Fig. 4C). To further explore this pathway, Huh7.5.1 cells were transfected with mCherry-2×-Rhotekin-GBD plasmids for 24 h, serving as a probe for GTP-RhoA. Following treatment with CQD, the ROCK1 inhibitor dasabuvir [25, 26], and a combination of both, we performed immunofluorescence analysis 2 h post-treatment. The results indicated a significant increase in the mCherry-2×-Rhotekin-GBD signal in CQD-treated cells, which was subsequently mitigated by dasabuvir (Fig. 4D and E).

PI(4,5)P2 is essential for migrasome formation [16, 27]. To assess the levels of PI(4,5)P2 following CQD treatment, Huh7.5.1 cells were transfected with PH-PLCD1-GFP plasmids for 24 h to serve as a probe for PI(4,5)P2. Cells were then exposed to 10 µg/mL of CQD and analyzed at 5 and 20 h using immunofluorescence. We observed a significant increase in the PH-PLCD1 signal at both time points, which was diminished upon treatment with the lipid kinase PIP5K1A inhibitor, ISA-2011B (Fig. 4F and G).Furthermore, when we combined dasabuvir to inhibit ROCK1 and ISA-2011B to inhibit PIP5K1A in CQD-treated Huh7.5.1 cells, we observed a substantial decrease in the percentage of migrasome-bearing cells, the total number of migrasomes, and the length of retraction fibers compared to cells treated with CQD alone, as evidenced by the mCherry-TSPAN4 migrasome marker (Fig. 4H − K). These findings suggest that CQD enhances RhoA activity via ROCK1 and promotes PI(4,5)P2 synthesis through PIP5K1A.

### CQD protects mitochondria by inducing mitocytosis

**Fig. 5:**
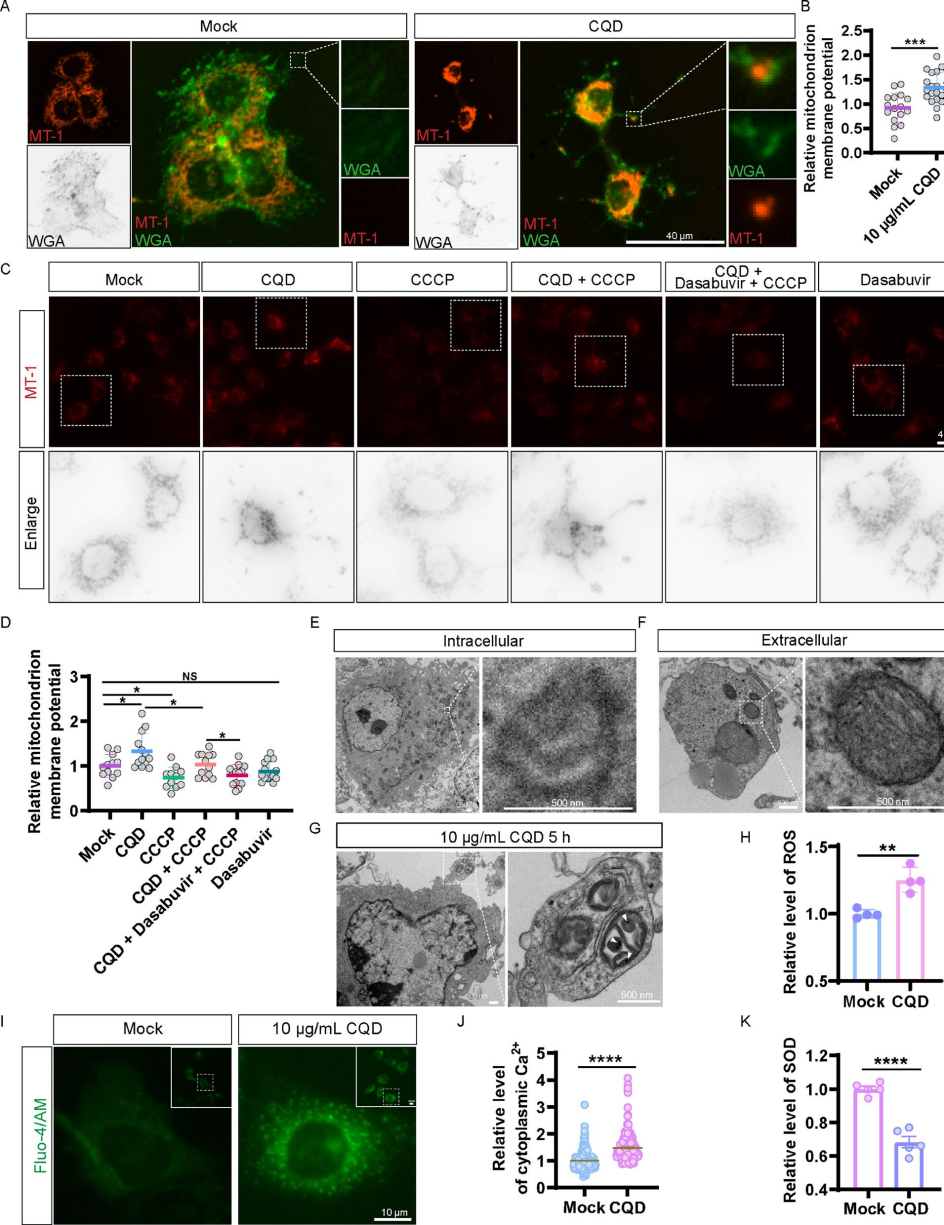
CQD upregulates ROS and cytoplasmic Ca²⁺, inhibits SOD enzyme activity, and promotes migrasome formation, thereby protecting MMP. (**A**) CQD protects mitochondrial integrity in Huh7.5.1 cells, and the colocalization of migrasome and MMP in enlarged panel. Scale bar: 40 μm. (**B**) Statistical analysis of panel A. (**C**) CQD restores MMP inhibited by CCCP and diminished by dasabuvir. Scale bar: 40 μm. (**D**) Statistical analysis of panel C. (**E**) TEM images depicting healthy mitochondria with intact cristae in10 µg/mL CQD treated for 5 h. Scale bar: 500 nm. (**F**) TEM images showcasing mitochondria exhibiting damaged cristae within migrasomes after 10 µg/mL CQD treated for 5 h. Scale bar: 500 nm. (**G**) TEM images illustrating the process of migrasome formation involving the discharge of damaged mitochondria from Huh7.5.1 cells treated with 10 µg/mL CQD for 5 h. The white triangle indicates CQD located within a damaged mitochondrion inside a migrasome. Scale bar: 500 nm. (**H**) 10 µg/mL CQD upregulates ROS. (**I**) 10 µg/mL CQD upregulates cytoplasmic Ca²⁺ level in Huh7.5.1 cells. (**J**) Statistical analysis of panel I. (**K**) 10 µg/mL CQD inhibit SOD enzyme activity. NS denotes *p* > 0.05; *, *p* < 0.05; **, *p* < 0.01; ***, *p* < 0.001; ****, *p* < 0.0001

Migrasomes have been characterized as organelles capable of transporting mitochondria [11]. To investigate the relationship between CQD, migrasomes, and mitochondria, we treated Huh7.5.1 cells with 10 µg/mL of CQD for 17 h, followed by staining with WGA and MT-1 (Fig. 5A). Analysis of fluorescence intensity revealed that CQD treatment significantly enhanced mitochondrial membrane potential compared to the control group (Mock) (Fig. 5B).

To further examine the protective role of CQD against mitochondrial damage, we first pre-treated the cells with 4 µM CCCP for 17 h to disrupt the mitochondrial membrane potential in Huh7.5.1 cells subsequently treated with 10 µg/mL of CQD. Our results demonstrated that CQD treatment was able to reverse the mitochondrial membrane potential impairment induced by CCCP.However, this protective effect was inhibited by the ROCK1 inhibitor dasabuvir (Fig. 5C and D). These findings suggest that 10 µg/mL of CQD may safeguard mitochondrial function by promoting RhoA-mediated migrasome formation, facilitating the transport of damaged mitochondria to the extracellular space.To validate this hypothesis, we analyzed mitochondrial dynamics using TEM. We observed the process by which CQD-treated cells expel damaged mitochondria encapsulated within migrasomes (Fig. 5E). Notably, mitochondrial damage was more pronounced within the migrasomes than in the cell body (Fig. 5F and G). Collectively, these results indicate that CQD protects mitochondria by facilitating migrasome formation and the transport of damaged mitochondria.

When mitochondria are damaged, the production of reactive oxygen species (ROS) can become excessive, leading to oxidative stress, which further exacerbates mitochondrial damage. We found that CQD increases intracellular ROS levels (Fig. 5H).The activation of intracellular calcium ions is an important pathway for the formation of migrasomes. Hence, we assessed the impact of CQD on intracellular calcium ion levels. The results showed that CQD increases intracellular calcium ion levels (Fig. 5I and J).Superoxide dismutase (SOD) is an important antioxidant enzyme that neutralizes oxidative stress caused by ROS, thereby protecting mitochondria from damage. Our findings revealed that CQD reduces intracellular SOD activity, resulting in elevated ROS levels due to impaired clearance (Fig. 5K). Taken together, these results suggest that CQD may ameliorate mitochondrial damage by increasing intracellular calcium ion levels to promote migrasome formation, but it does not appear to do so by reducing ROS or enhancing SOD activity.

### GQD and GOQDs promote migrasome formation

**Fig. 6:**
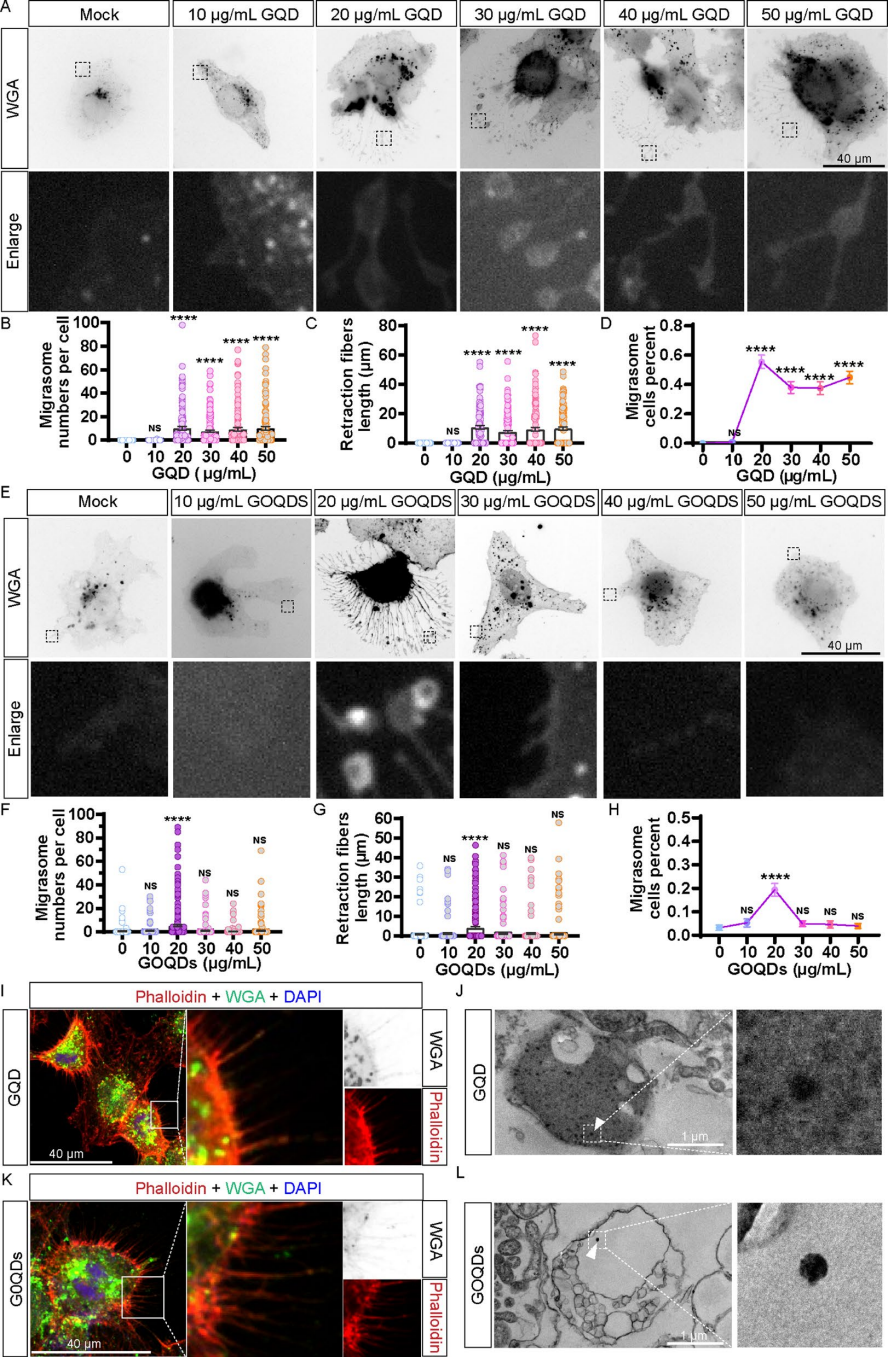
GQD and GOQDs induce migrasomes formation. (**A**) Huh7.5.1 cells were treated with varying concentrations of GQD (0 to 50 µg/mL) for 5 h. The images have been converted to gray-value mode for clarity. Scale bar: 40 μm. (**B** -**D**) Quantitative analysis of migrasome formation, including the number of migrasomes, retraction fiber lengths, and the proportion of migrasome-generating cells, as derived from the data presented in panel A. A minimum of 118 cells per group were analyzed across three independent experiments. **(E**) Huh7.5.1 cells were subjected to GQD treatment (0 to 50 µg/mL) for 5 h. Scale bar: 40 μm. (**F**–**H**) Statistical evaluation of migrasome quantity, retraction fiber lengths, and the percentage of migrasome-generating cells in panel E. (**I**) Confocal images of Huh7.5.1 cells treated with 20 µg/mL GQD for 17 h and stained by phalloidin for retraction fiber and WGA for migrasome. Scale bar: 40 μm. (**J**) TEM images revealing migrasomes in Huh7.5.1 cells treated with 20 µg/mL GQD for 17 h. A white triangle indicates GQD presence within migrasomes. Scale bar: 1 μm. (**K**) Images of Huh7.5.1 cells treated with 20 µg/mL GOQD for 17 h. Scale bar: 40 μm. (**L**) TEM images of migrasomes in Huh7.5.1 cells treated with 20 µg/mL GOQD for 17 h, highlighting the presence of GOQD within the migrasomes (white triangle). Scale bar: 1 μm. NS denotes *p* > 0.05; *, *p* < 0.05; ****, *p* < 0.0001

To investigate the effects of different carbon nanostructures on the cytoskeleton, we examined the influence of various concentrations of GQD on migrasome formation in Huh7.5.1 cells. Following a 5-h treatment with GQD, cells were stained with WGA to visualize migrasomes. We observed that concentrations ranging from 20 to 50 µg/mL effectively induced migrasome formation (Fig. 6A − D). In contrast, treatment with GOQDs at concentrations of 20 µg/mL resulted in only a slight promotion of migrasome formation (Fig. 6E − H). Notably, both GQD and GOQD treatments led to the induction of retraction fibers, as evidenced by phalloidin staining (Fig. 6I and J). These results underscore the differential effects of carbon nanostructures on cytoskeletal dynamics and migrasome formation in Huh7.5.1 cells. In contrast, 5 nm gold particles of similar size showed no significant impact on migrasome formation after 5 h of treatment (Fig. S4A and S4B). Using TEM, we observed the localization of GQD within the migrasomes induced by GQD treatment in Huh7.5.1 cells (Fig. 6K). Similarly, TEM results revealed the presence of GOQDs within the migrasomes induced by GOQDs treatment in Huh7.5.1 cells (Fig. 6L).

## GQD upregulates the levels of GTP-RhoA, cholesterol and PI(4,5)P2

**Fig. 7:**
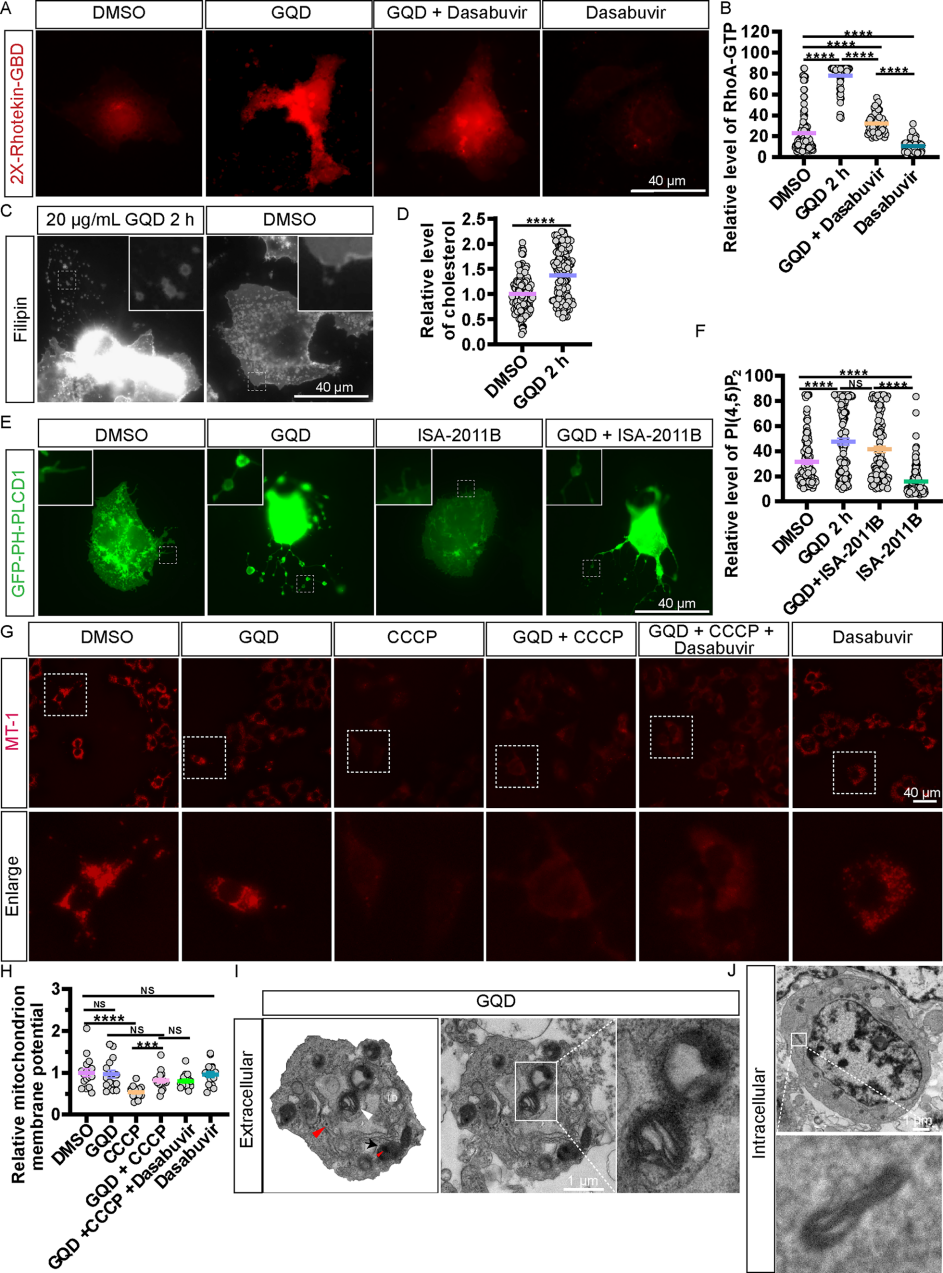
GQD upregulates active RhoA, cholesterol, and PI(4,5)P2, protecting against CCCP-induced mitochondrial damage. (**A**) Huh7.5.1 cells expressing mCherry-2×-Rho-TEK-GBD were treated with or without 20 µg/mL GQD, 15 µM dasabuvir, or a combination of GQD and dasabuvir for 2 h. Scale bar: 40 μm. (**B**) Quantitative analysis of fluorescence intensity in panel A. (**C**) Huh7.5.1 cells stained with filipin were treated with or without GQD for 2 h. Scale bar: 40 μm. (**D**) Statistical analysis of fluorescence intensity in panel C. (E) Huh7.5.1 cells expressing PH-GFP-PLCD-1 were treated with or without 20 µg/mL GQD, 100 µM ISA-2011B, or their combination for 2 h. Scale bar: 40 μm. (**F**) Statistical evaluation of fluorescence intensity in panel E. (**G**) GQD counters CCCP-induced loss of mitochondrial membrane potential in Huh7.5.1 cells. Scale bar: 40 μm. (**H**) Relative fluorescence intensity analysis in panel G. (**I**) TEM images depicting migrasomes containing mitochondria, lysosomes, and lipid droplets in Huh7.5.1 cells treated with 20 µg/mL GQD for 17 h. White triangle indicates mitochondria, LD indicates lipid droplets, red triangle denotes GQD, and black arrow points to lysosomes within migrasomes. (**J**) TEM images of Huh7.5.1 cells depicting swollen mitochondrial ridges after 17 h of treatment with 20 µg/mL GQD. Scale bar: 1 μm. NS denotes *p* > 0.05; **, *p* < 0.01; ***, *p* < 0.001; ****, *p* < 0.0001

To investigate whether GQD influences RhoA activation, we transfected Huh7.5.1 cells with mCherry-2×-Rhotekin-GBD plasmids. After treating the cells with 20 µg/mL of GQD, dasabuvir, or a combination of both for 2 h, we performed immunofluorescence analysis. The results revealed a significant increase in the 2×-Rhotekin-GBD signal in GQD-treated cells, an effect that was effectively inhibited by dasabuvir (Fig. 7A and B). Subsequently, Huh7.5.1 cells were treated with 20 µg/mL of GQD for 2 h, followed by staining with filipin to visualize cholesterol. The results indicated a significant increase in cholesterol levels in the GQD-treated cells compared to the control group (Fig. 7C and D). Additionally, Huh7.5.1 cells were transfected for 24 h with PH-PLCD1-GFP plasmids and treated with 20 µg/mL of GQD for 2 h. Immunofluorescence analysis revealed a notable enhancement in the PH-PLCD1 signal, which was subsequently diminished upon treatment with the lipid kinase PIP5K1A inhibitor, ISA-2011B, resulting in reduced levels of PI(4,5)P2 (Fig. 7E and F). Taken together, GQD could upregulate the levels of GTP-RhoA, cholesterol and PI(4,5)P2.

### Effects of GQD on mitochondrial integrity

To investigate the relationship between GQD and mitochondria, Huh7.5.1 cells were treated with 20 µg/mL of GQD for 17 h, followed by staining with MT-1 to visualize mitochondrial dynamics. Cells pre-treated with 4 µM CCCP for 17 h to disrupt mitochondrial membrane potential exhibited a reversal of the damage induced by CCCP when subsequently treated with GQD (Fig. 7G and H). Next, we analyzed mitochondrial morphology using TEM. Our observations revealed swollen mitochondria exhibiting unfolded cristae in GQD-treated Huh7.5.1 cells and migrasomes, consistent with the changes in mitochondrial membrane potential (Fig. 7I and J).

### GOQDs upregulate the levels of active RhoA, cholesterol and PI(4,5)P2

**Fig. 8:**
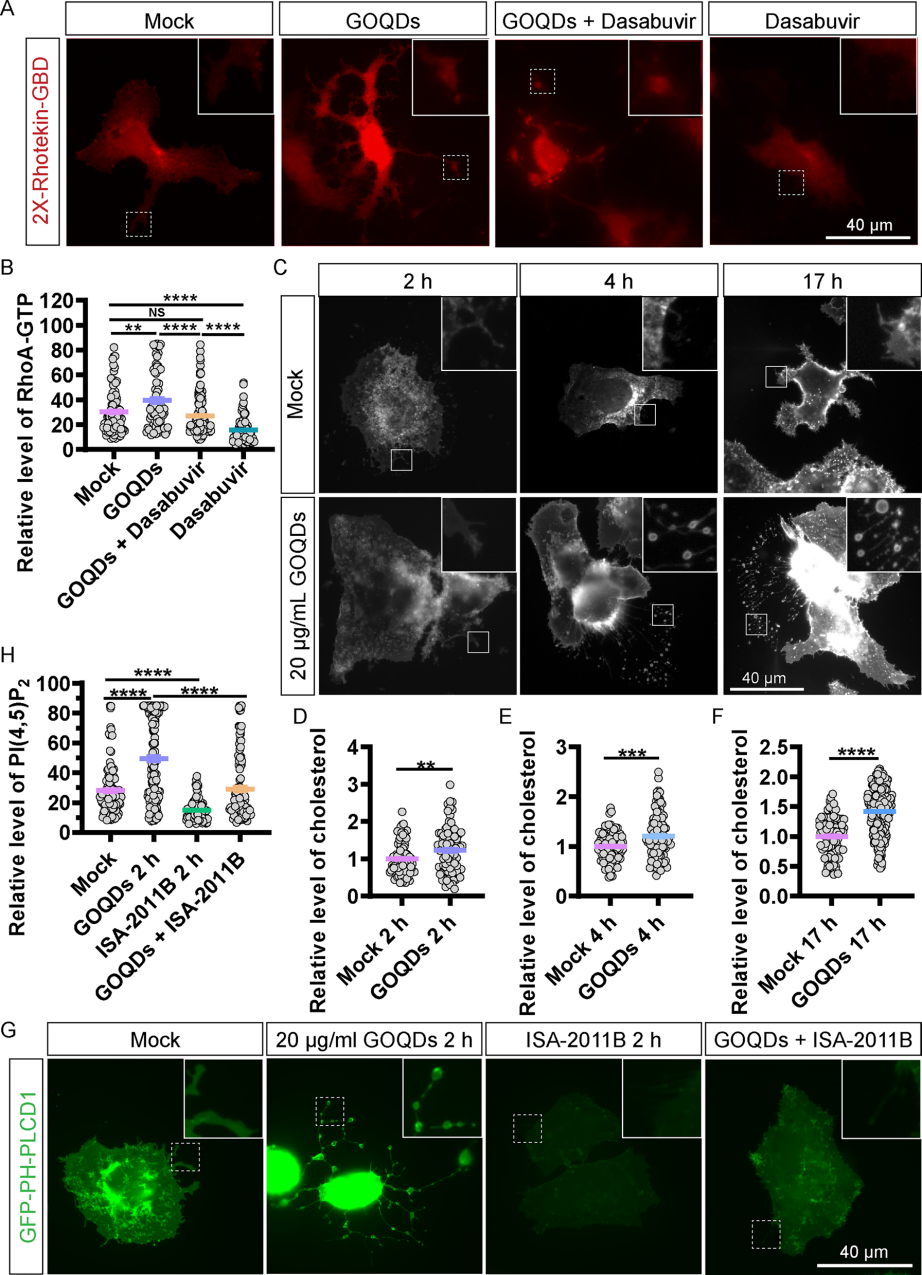
GOQDs upregulate active RhoA, cholesterol, and PI(4,5)P2. (**A**) Huh7.5.1 cells expressing mCherry-2×-Rho-TEK-GBD were treated with or without 20 µg/mL GOQDs, 15 µM dasabuvir, or their combination for 2 h. Scale bar: 40 μm. (**B**) Statistical analysis of panel A. (**C**) Huh7.5.1 cells stained with filipin. Scale bar: 40 μm. (**D **– **F**) Statistical analysis of panel C. (**G**) Huh7.5.1 cells expressing PH-GFP-PLCD-1 were treated with or without GOQDs, 100 µM ISA-2011B, or a combination of these for 2 h. Scale bar: 40 μm. (**H**) Statistical analysis of fluorescence intensity in G

Notably, treatment with GOQDs significantly enhanced the activity of RhoA marked by 2×-Rhotekin-GBD signal, which was subsequently inhibited by dasabuvir after 2 h of exposure (Fig. 8A and B). Furthermore, the levels of cholesterol stained by filipin were elevated following treatment with 20 µg/mL of GOQDs, with this increase observed as early as 2 h and continuing to improve over time (Fig. 8C – F). Immunofluorescence analysis indicated that treatment with 20 µg/mL of GOQDs for 2 h resulted in a significant enhancement in the PH-PLCD1 signal, indicating the upregulation of PI(4,5)P2 (Fig. 8G and H). However, this increase was attenuated upon the application of the lipid kinase PIP5K1A inhibitor, ISA-2011B, which leads to a reduction in PI(4,5)P2 levels (Fig. 8G and H).

### Effects of GOQDs on mitochondrial integrity

**Fig. 9:**
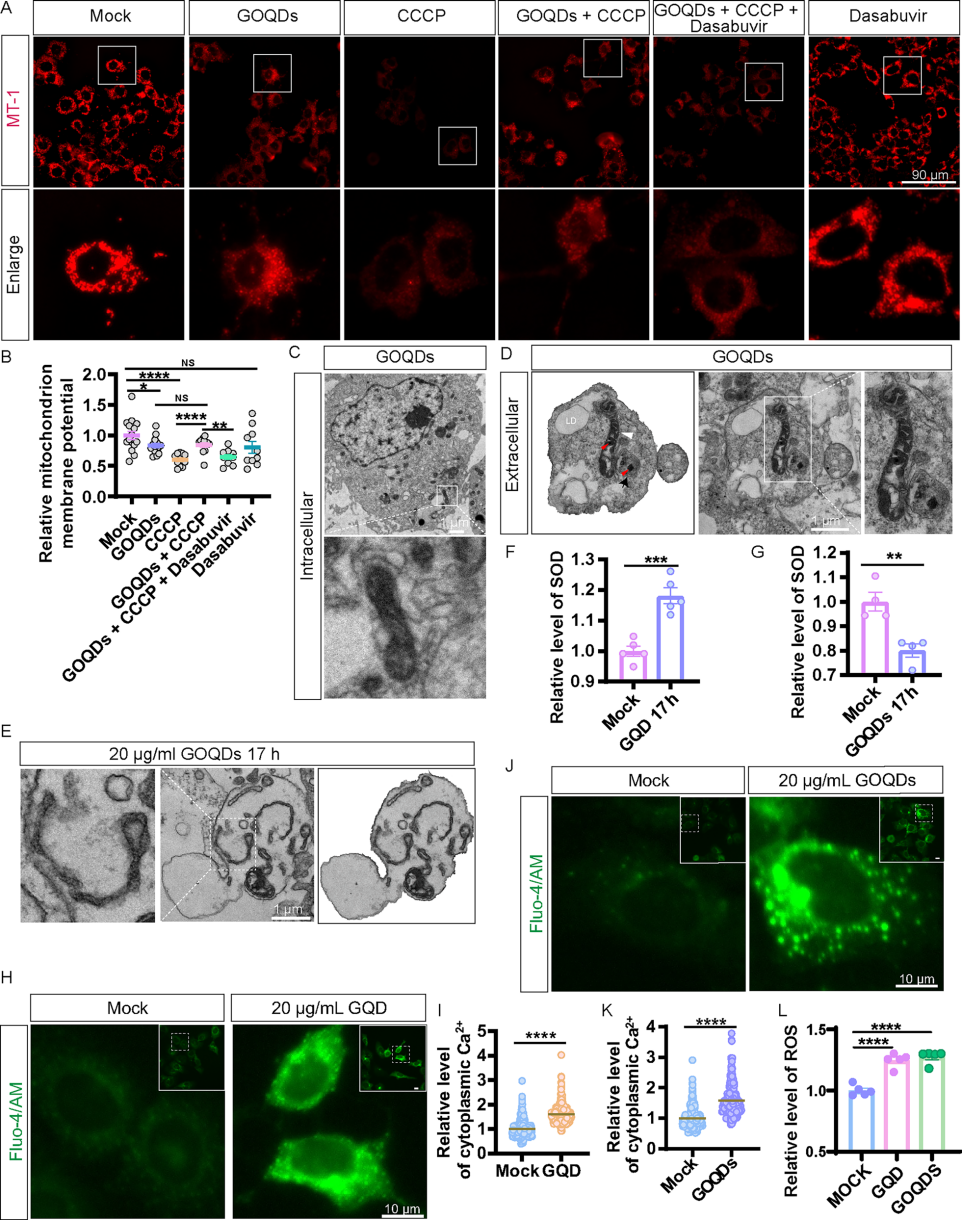
GOQDs protect against CCCP-induced mitochondrial damage by regulating ROS, cytoplasmic Ca²⁺, and SOD enzyme activity. (**A**) Examination of mitochondrial membrane potential damage in Huh7.5.1 cells treated with GOQDs (20 µg/mL), CCCP (4 µM), dasabuvir (15 µM), GOQDs + CCCP, and GOQDs + CCCP + dasabuvir for 17 h. Scale bar: 90 μm. (**B**) Statistical evaluation of panel A. (**C**) TEM images indicating mitochondrial pyknosis in Huh7.5.1 cells treated with 20 µg/mL GOQDs for 17 h. Scale bar: 1 μm. (**D**) TEM images of migrasomes containing mitochondria, lysosomes, and lipid droplets in Huh7.5.1 cells treated with 20 µg/mL GOQDs for 17 h. White triangle denotes mitochondria, LD indicates lipid droplets, red triangle marks GOQDs within migrasomes, and black arrow points to lysosomes. Scale bar: 1 μm. (**E**) TEM images of endoplasmic reticulum structures within migrasomes. Scale bar: 1 μm. (**F**) GQD upregulates SOD enzyme activity. (**G**) GOQDs inhibit SOD enzyme activity. (**H**) GQD upregulates cytoplasmic Ca²⁺ level in Huh7.5.1 cells. (**I**) Statistical analysis of panel H. (**J**) GOQDs upregulates cytoplasmic Ca²**⁺** level in Huh7.5.1 cells. (**K**) Statistical analysis of panel J. (**L**) GQD and GOQDs upregulates ROS. NS denotes *p* > 0.05; *, *p* < 0.05; **, *p* < 0.01; ***, *p* < 0.001; ****, *p* < 0.0001

To explore the connection between GOQDs and mitochondria, Huh7.5.1 cells were treated with 20 µg/mL of GOQDs for 17 h, followed by staining with MT-1. Analysis of fluorescence intensity revealed that GOQDs disrupted mitochondrial membrane potential compared to the control group (Mock) (Fig. 9A and B). Additionally, the cells pre-treated with 4 µM CCCP for 17 h to disrupt mitochondrial membrane potential and then treated with 20 µg/mL of GOQDs could reverse the mitochondrial membrane potential damage induced by CCCP. However, this protective effect was inhibited by the ROCK1 inhibitor dasabuvir (Fig. 9A and B). These findings suggest that both 20 µg/mL of GOQDs may protect mitochondria by promoting RhoA-mediated migrasome formation, thereby facilitating the transport of damaged mitochondria to the extracellular space. To validate this hypothesis, we conducted TEM analyses of mitochondrial morphology. Mitochondria in GOQD-treated Huh7.5.1 cells exhibited a more severe state of pyknosis than GQD treatment (Fig. 9C), consistent with the observed alterations in mitochondrial membrane potential. Interestingly, migrasomes contained mitochondria, lysosomes, lipid droplets, GOQDs, and endoplasmic reticulum in Huh7.5.1 cells treated with 20 µg/mL GOQDs for 17 h (Fig. 9D and E).

Next, we investigated how GQD and GOQDs affected SOD, ROS, and intracellular calcium ion levels. GQD increased the activity of intracellular SOD (Fig. 9F), while GOQDs significantly inhibited intracellular SOD activity (Fig. 9G). Both GQD and GOQDs elevated calcium ion levels in the cytoplasm (Fig. 9H − 9 K). Additionally, both GQD and GOQDs were found to increase ROS levels (Fig. 9L). We conclude that GQD and GOQDs can alleviate mitochondrial damage by increasing calcium ion concentrations, thereby promoting migrasome formation.

### CQD, GQD and GOQDs increase migrasome numbers in mouse blood

**Fig. 10:**
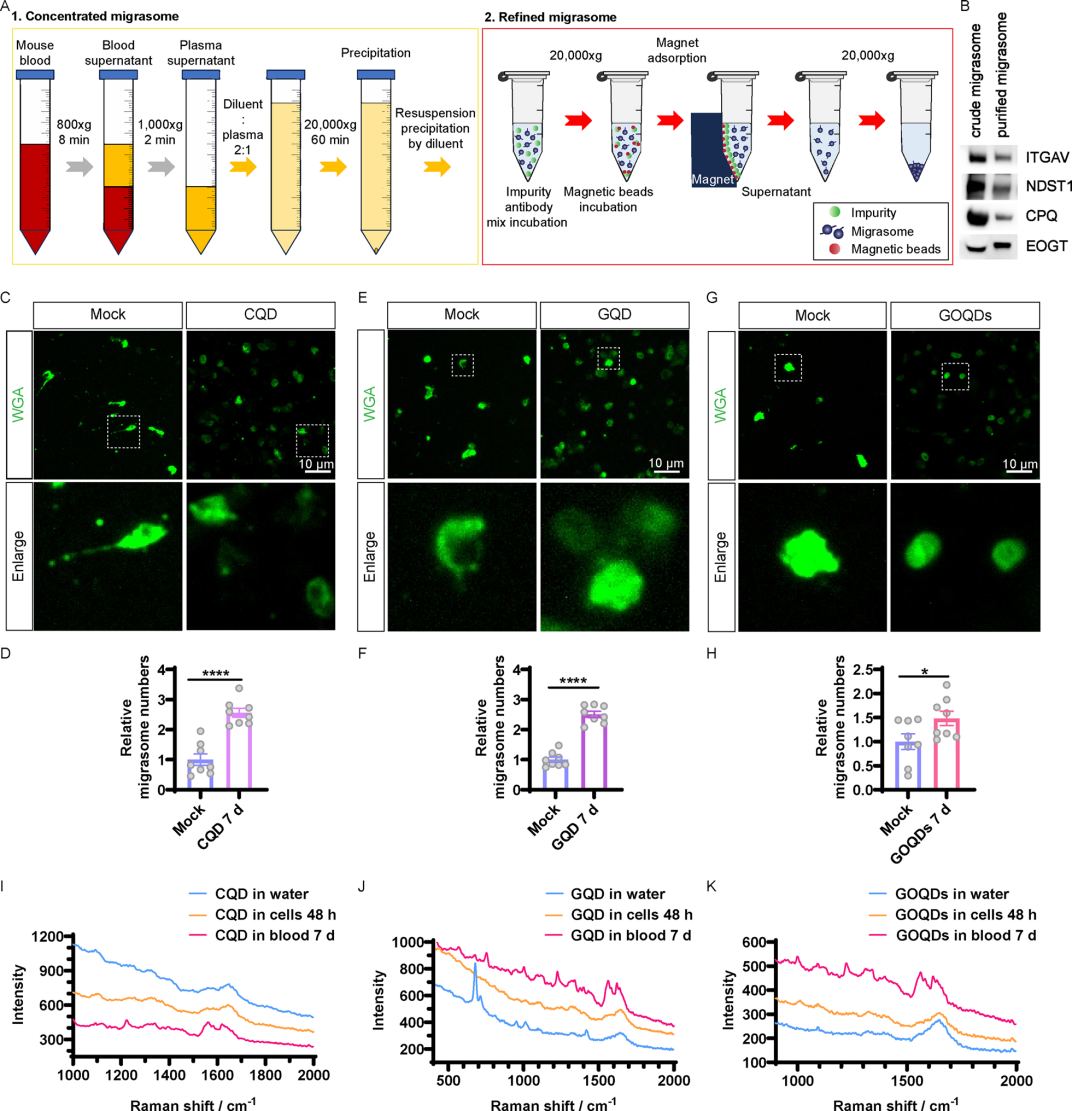
Effects of CQD, GQD and GOQDs on migrasomes in mice. (**A**) The diagram illustrates the migrasome purification protocol in mouse blood. Step 1 involves obtaining crude migrasomes through concentration. Step 2 focuses on isolating refined migrasomes using the Migralso™ Pro Mouse Immune Cell Extraction Kit. (**B**) The purification of migrasomes was confirmed by Western blotting using specific migrasome markers. (**C**) CQD increased migrasomes in the blood of mice. Scale bar: 10 μm. (**D**) Statistical analysis of panel C. (**E**) GQD treatment increased the number of migrasomes in the blood of mice. Scale bar: 10 μm. (**F**) Statistical analysis of E. (G) GOQDs increased migrasomes in the blood of mice. Scale bar: 10 μm. (**H**) Statistical analysis of G. (**I**) Raman spectrum of CQD in water, in cells after 48 h, and in blood after 7 days. (**J**) Raman spectrum of GQD in water, in cells after 48 h, and in blood after 7 days. (**K**) Raman spectrum of GOQDs in water, in cells after 48 h, and in blood after 7 days

Next, we investigate the effects of CQD, GQD and GOQDs on the numbers of migrasomes in vivo. The crude migrasomes from mouse whole blood were isolated as shown in step 1 of Fig. 10A. The refined migrasomes were extracted using Migralso™ Pro Mouse Immune Cells extraction kit as shown in step 2 of Fig. 10A. The migrasome markers CPQ, EGOT, ITGAV and NDST1 were detected by Western blots (Fig. 10B). We isolated whole blood migrasomes from mice that were intraperitoneally injected with 3 µg/g body weight of CQD or physiological saline on the seventh day post-injection. The administered doses of CQD have been confirmed to be non-toxic to the liver and kidneys of the mice (Fig. S5A, S5B, S5C and S5D). Our analysis revealed that CQD significantly increased the number of migrasomes in the mice’s blood (Fig. 10C and D).

Additionally, we isolated whole blood migrasomes from another group of mice injected with 6 µg/g body weight of GQD or physiological saline, also on the seventh day after injection. The doses of GQD administered have been verified to be non-toxic to the liver and kidneys of the mice (Fig. S5A, S5B, S5C and S5D). The results indicated that GQD treatment led to a notable increase in migrasome numbers in the blood (Fig. 10E and F).

Furthermore, we conducted a similar isolation from mice that received 6 µg/g body weight of GOQDs or physiological saline. The administered doses of GOQD have been confirmed to pose no toxicity to the liver and kidneys of the mice (Fig. S5A, S5B, S5C and S5D). The findings demonstrated that the presence of GOQDs was associated with a higher count of migrasomes in the blood of these mice (Fig. 10G and H).

To elucidate the chemical stability of the QDs and their oxidation status, we conducted FT-Raman measurements to analyze the QDs both over a 48-hour timeframe within the cells and from whole blood collected from the mice. As shown in Fig. 10I − K, CQD, GQD and GOQDs were detectable in both the cells treated with QDs for 48 h and the mice treated with QDs for 7 days.

### CQD, GQD and GOQDs increase platelet counts, platelet aggregation, and bleeding volumes in mice

**Fig. 11:**
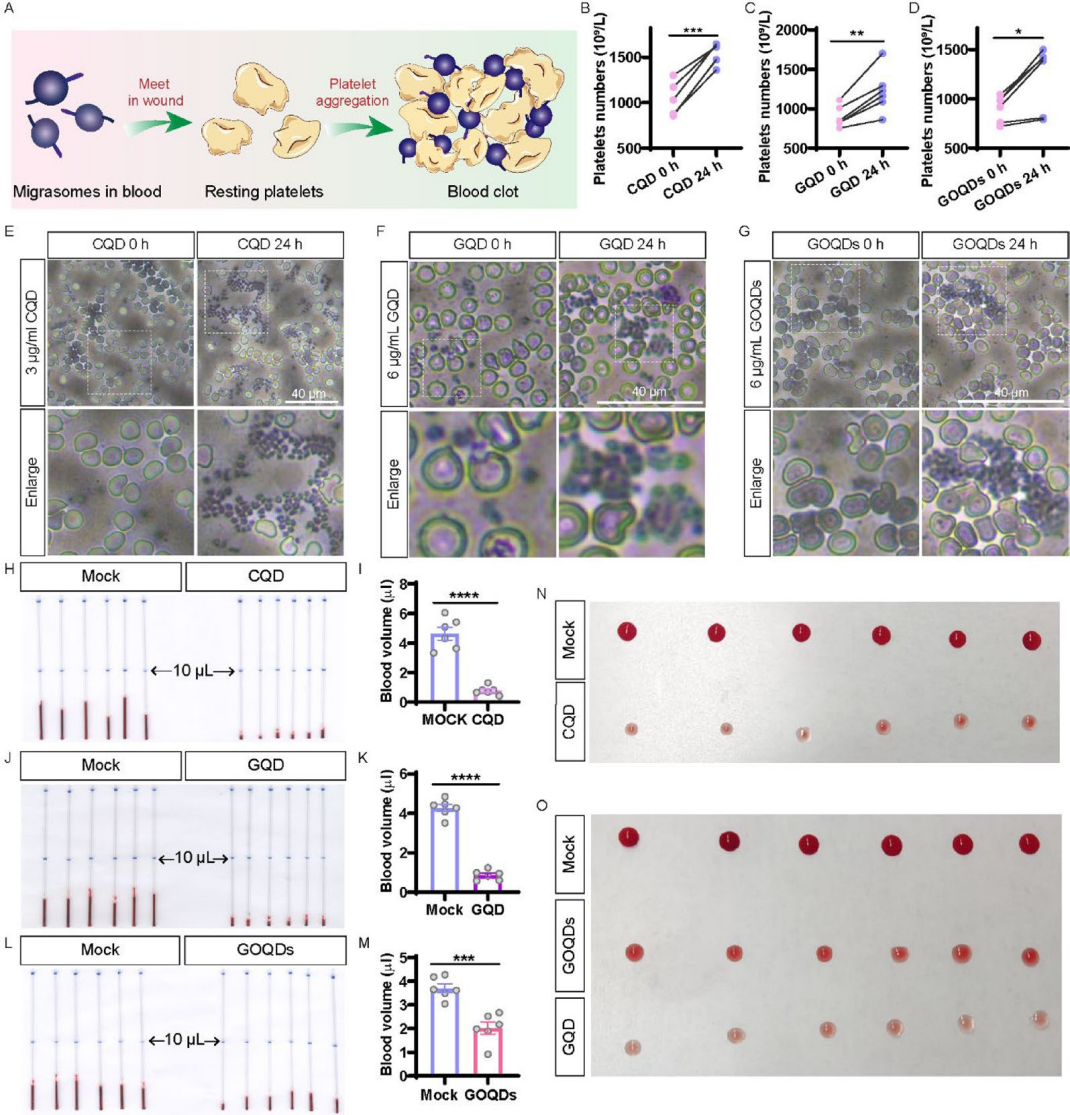
Effects of CQD, GQD and GOQDs on platelet dynamics in mice. (**A**) Diagram illustrating the impact of migrasomes in blood on platelet function and coagulation dynamics. (**B**) Statistical analysis of platelet numbers in mouse blood, after treated with CQD for 0 and 24 h. (**C**) Platelet numbers treated with GQD for 0 and 24 h. (**D**) Platelet numbers treated with GOQDs for 0 and 24 h. (**E**) Images of aggregated platelets from blood smears of mice treated with CQD. (**F**) Aggregated platelets after treated with GQD. (G) Aggregated platelets after treated with GOQDs. (**H**) Assessment of peripheral vascular bleeding in mice treated with CQD for 24 h. (**I**) Statistical analysis of H. (**J**) Assessment of peripheral vascular bleeding in mice treated with GQD for 24 h. (**K**) Statistical analysis of J. (**L**) Assessment of peripheral vascular bleeding in mice treated with GOQDs for 24 h. (M) Statistical analysis of L. (**N**) Comparison of peripheral vascular bleeding in mice treated with CQD for 24 h versus untreated mice. (**O**) Comparison of peripheral vascular bleeding in mice treated with GQD or GOQDs for 24 h versus untreated mice. *, *p* < 0.05; **, *p* < 0.01; ***, *p* < 0.001; ****, *p* < 0.0001

Previous study has demonstrated that an increase in migrasomes within the bloodstream can affect platelet count and function by promoting the secretion of platelets from neutrophils [28]. Thus, increased migrasome formation by CQD, GQD and GOQDs potentially enhances platelet production and activation, which ultimately influences coagulation function (Fig. 11A). To investigate the effects of CQD, GQD and GOQDs on platelet count and function in vivo, we evaluated their influence on migrasomes. Mice received an intraperitoneal dose of 3 µg of CQD per gram of body weight, 6 µg of GQD per gram of body weight, 6 µg of GOQDs per gram of body weight. 24 h after treatment, blood samples were diluted with a platelet dilution buffer for platelet counting using a hemocytometer. Blood smears were prepared and stained with Wright’s stain to assess platelet aggregation. The results revealed a significant increase in both platelet count (Fig. 11B – D) and platelet aggregation (Fig. 11E – G) compared to baseline levels. Bleeding volumes were significantly reduced in mice treated with CQD, GQD and GOQDs over the 24-h observation period (Fig. 11H − O). These findings suggest that CQD, GQD and GOQD enhance platelet production and activation, and ultimately influencing hemostatic function.

## Discussion

**Fig. 12:**
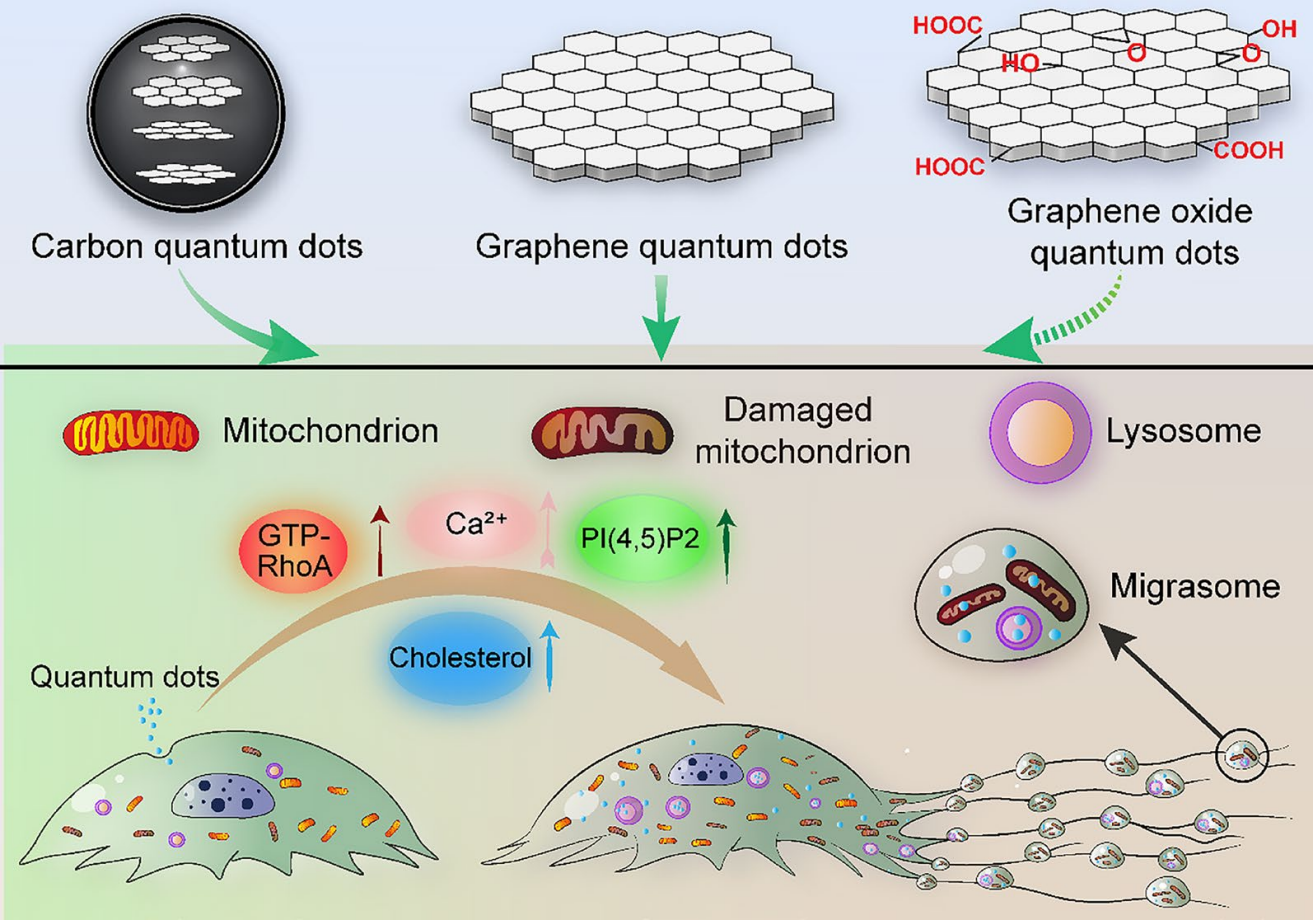
The model of the role of CQD, GQD and GOQDs in migrasome formation

The current study sheds light on the unique biological effects of carbon-based quantum dots, specifically CQD, GQD, and GOQDs. We emphasize their influence on migrasome formation, mitochondrial integrity, and the regulation of platelet function (Fig. 12). Our findings indicate that these nanoparticles substantially affect cellular dynamics through various mechanisms, including the activation of RhoA signaling pathway and the enhancement of cellular levels of cholesterol, PI(4,5)P2 and Ca^2+^ (Fig. 12).

In prior investigations, we discovered that nanoparticles from 28 nm to 80 nm, regardless of whether they were microplastics or metal oxides [17, 18], could induce migrasome formation. Moreover, we observed a direct correlation between particle diameter and the number of migrasomes formed at the same concentration. To explore this further, our current study examined smaller nanoparticles (less than 10 nm), focusing on carbon quantum dots, which are renowned for their excellent water solubility, low toxicity, environmental safety, and high biocompatibility. Despite the rapid technological advancements of carbon quantum dots, research into their cellular impacts remains limited. These materials are exceptionally chemically stable in both acidic and alkaline environments and can maintain their structural integrity once internalized by cellular organelles, thereby resisting degradation by lysosomes or mitochondria. This stability allows for the tracking of their metabolic processes within cells.

The carbon quantum dot family comprises traditional CQD, GQD, carbon polymer dots (CPD), and carbon nanodots (CND), among various carbon composites [29]. GQD usually originated from graphene or graphite, forming highly crystalline graphene nanosheets with a disk-like structure. In contrast, CQD consists of layered graphene nanosheets that preserve the lattice structure and high crystallinity of graphene, incorporating heteroatoms in their cores and abundant functional groups along their edges. Our studies demonstrated that CQD less than 10 nm in diameter can indeed promote migrasome formation, whereas 5 nm gold nanomaterials do not. TEM studies revealed that upon entering lysosomes and mitochondria, cells can expel damaged organelles via migrasomes to maintain normal physiological functions. We also investigated how different nanoparticle structures of similar size impact migrasome formation. Notably, while CQD only induced migrasome formation at concentrations ranging from 10 µg/mL to 30 µg/mL, GQD stimulated migrasome generation between 20 µg/mL and 50 µg/mL, and possibly at even higher concentrations. Interestingly, GOQDs stimulated migrasome formation only at approximately 20 µg/mL. This indicates that structural variations can lead to differing effects on migrasome development.

We observed that the average sizes of GQD, CQD, and GOQDs are 4.363 ± 1.927 nm, 4.091 ± 0.9755 nm, and 2.617 ± 0.6097 nm, respectively, which correlate with the concentration ranges required to promote migrasome formation. Notably, smaller nanoparticles exhibited a narrower concentration range for promoting migrasome formation. A similar phenomenon was observed in a previous study on ZnO-NPs. In that study, while 28 nm ZnO NPs only induced migrasome formation at concentrations ranging from 10 µg/mL to 30 µg/mL, 36 nm ZnO NPs stimulated migrasome generation between 10 µg/mL and 50 µg/mL, and possibly at even higher concentrations.

Previous work indicated that all nanoparticles within the size range of 2–100 nm can alter the signaling processes essential for fundamental cellular functions, with 40 nm and 50 nm nanoparticles exhibiting the most pronounced effects [30, 31]. Therefore, nanoparticles should no longer be regarded merely as passive carriers for biomedical applications; instead, they can actively mediate biological effects. We previously discovered that 28 nm ZO-NPs, 36 nm ZO-NPs, and 80 nm PS-NPs could induce migrasome formation [17, 18]. Our current study has found that carbon-based ultrasmall materials smaller than 10 nm can also induce migrasome formation, thereby extending the minimum size required for inducing migrasome formation. This effect is likely due to the π-conjugated carbon cores and abundant surface functional groups of CQD, GQD, and GOQDs, which facilitate stronger interactions with cellular membranes and mitochondria.

Further exploration into the mechanisms of migrasome formation showed that all three types of materials activated RhoA, with GQD exhibiting a more significant activation level compared to GOQDs. The application of the RhoA inhibitor dasabuvir completely counteracted the activation induced by GOQDs, though it only partially reduced the effects of GQD. Additionally, we observed that all three materials upregulated the level of cholesterol, an essential component of plasma membranes. In experiments with GOQDs, we noted a gradual increase in cholesterol levels peaking at 17 h, while GQD significantly elevated membrane cholesterol levels as early as 2 h. Both GQD and GOQDs activated PI(4,5)P2 at the two-hour mark, yet the effect of GQD remained unaffected by the PI(4,5)P2 inhibitor ISA2011.

Our findings indicate that CQD, GQD and GOQDs play a protective role against mitochondrial damage by facilitating the expulsion of injured mitochondria encapsulated in migrasomes. This novel mechanism suggests that these carbon nanostructures may promote cellular health, a notion particularly relevant in cancer biology where mitochondrial dysfunction significantly contributes to disease progression. Additionally, the ability of CQD, GQD, and GOQDs to enhance platelet count and aggregation aligns with previous research on the role of migrasomes in hemostatic functions [28]. This observation may inform therapeutic strategies for conditions like thrombocytopenia, where promoting blood coagulation is beneficial.

The relief of mitochondrial damage by three QDs cannot be solely explained by mitocytosis. Regulating autophagy-related pathways can also mitigate mitochondrial damage. For instance, activating the PINK1-PRKN pathway or inhibiting cholesterol accumulation can restore mitochondrial autophagy function [32]. Research suggests that GQD can induce autophagy, underscoring their potential to enhance cellular degradation and recycling processes [33–35]. This induction may contribute to various biological effects associated with GQD treatment, making them particularly relevant in the study of autophagy regulation. In contrast, studies indicate that GOQDs inhibit autophagy [36]. This inhibitory effect raises concerns about cellular health and may impact the therapeutic applications of GOQDs, especially in contexts where autophagy is crucial for maintaining cellular homeostasis and responding to stress. Currently, there is insufficient research on the role of CQD in autophagy, leaving their effects unclear and necessitating further investigation to elucidate their influence on this critical cellular process.

## Conclusion

In summary, our studies significantly improve our understanding of the role of nanoparticles smaller than 10 nm, specifically CQD, GQD, and GOQDs, in the formation of migrasomes. Our findings demonstrate that these nanoparticles notably enhance migrasome formation and safeguard mitochondrial integrity by modulating key signaling pathways involving RhoA, as well as Ca^2+^, cholesterol and PI(4,5)P2 levels. This underscores their capacity to influence cellular responses to environmental challenges. Furthermore, our results reveal the impact of these nanostructures on platelet activity, emphasizing their potential to improve wound healing processes. Ultimately, this research underscores the important roles of CQD, GQD, and GOQDs in cellular dynamics, paving the way for future applications in both biomedicine and the mitigation of environmental hazards.

## Supplementary Information

Below is the link to the electronic supplementary material.

Supplementary Material 1

Supplementary Material 2

Supplementary Material 3

Supplementary Material 4

Supplementary Material 5

## Data Availability

All data generated in this study are available from the corresponding author.

## Abbreviations

CQD: Carbon quantum dots
GQD: Graphene quantum dots
GOQDs: Graphene oxide quantum dots
ROCK1: Rho-associated protein kinase 1
PI(4,5)P2: Phosphatidylinositol-4,5-bisphosphate
PBS: Phosphate-buffered saline
DMSO: Dimethyl sulfoxide
FT-IR: Fourier transform infrared
DLS: The dynamic light scattering
PDI: Polydispersity index
PH-PLCD1-GFP: GFP-tagged PH domain of phospholipase C delta 1
CCCP: Carbonyl cyanide 3-chlorophenylhydrazone
DEME: Dulbecco’s modified Eagle’s medium
FBS: Fetal bovine serum
CCK-8: Cell counting kit-8
ROI: Region of interest
HRTEM: High resolution transmission electron microscopy
TEM: Ttransmission electron microscopy
ROS: Reactive oxygen species
SOD: Superoxide dismutase
OD: Optical density
CPD: Carbon polymer dots
CND: Carbon nanodots
