# Supplementary material for: Carbon-based quantum dots enhance platelets aggregation through migrasomes biogenesis
Source: J Nanobiotechnology. 2026 Jan 17;24:152. doi: 10.1186/s12951-025-04010-9 (PMC12896335; doi:10.1186/s12951-025-04010-9)
Supplement: Supplementary file 1 — Supplementary Material 1 [file 12951_2025_4010_MOESM1_ESM.pdf]

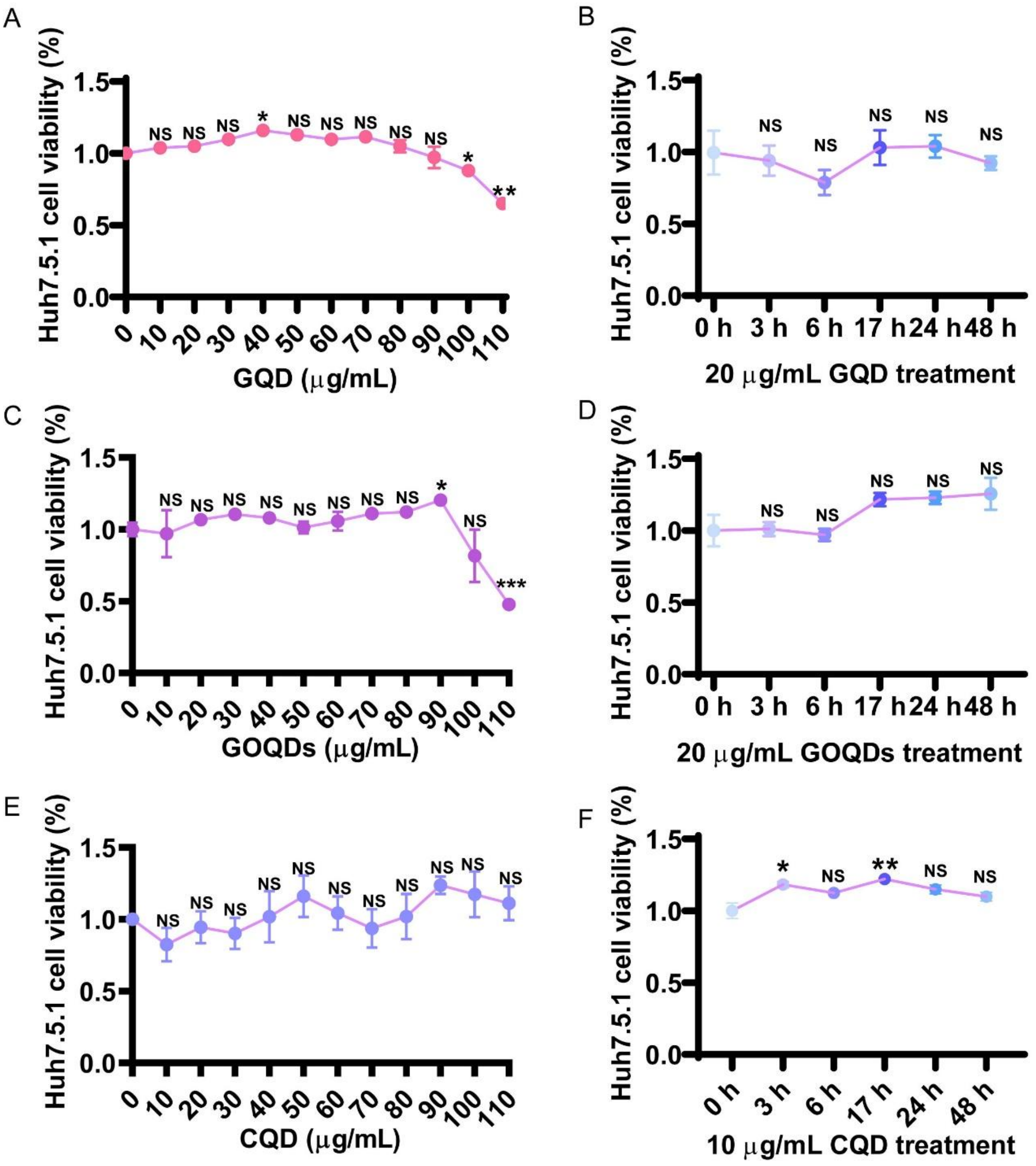

Fig. S1. Cell viability of Huh7.5.1 cells treated with GQD, GOQDs and CQD. (A) Cell viability of Huh7.5.1 cells treated with concentrations ranging from 0 to 110 μg/mL of GQD for 17 hours, as assessed by CCK-8 assays. Values are derived from three independent experiments. (B) Cell viability of Huh7.5.1 cells treated with 20 μg/mL of GQD over a time range of 0 to 48 hours, assessed by CCK-8 assays. Values from five independent experiments. (C) Cell viability of Huh7.5.1 cells treated with 0 to 110 μg/mL of GOQDs for 17 hours, evaluated using CCK-8 assays. Values are derived from three independent experiments. (D) Cell viability of Huh7.5.1 cells treated with 20 μg/mL of GOQDs over 0 to 48 hours, as assessed by CCK-8

assays. Values from five independent experiments. (E) Cell viability of Huh7.5.1 cells treated with 0 to 110 μg/mL of CQD for 17 hours, as determined by CCK-8 assays. Values are derived from three independent experiments. (F) Cell viability of Huh7.5.1 cells treated with 30 μg/mL of CQD over a period of 0 to 48 hours, as determined by CCK-8 assays. Values from five independent experiments. All data are reported as means ± SEM. Statistical significance was assessed using a two-tailed unpaired t-test, where NS indicates $p > 0.05$; * $p < 0.05$; ** $p < 0.01$.
