# Supplementary material for: Carbon-based quantum dots enhance platelets aggregation through migrasomes biogenesis
Source: J Nanobiotechnology. 2026 Jan 17;24:152. doi: 10.1186/s12951-025-04010-9 (PMC12896335; doi:10.1186/s12951-025-04010-9)
Supplement: Supplementary file 2 — Supplementary Material 2 [file 12951_2025_4010_MOESM2_ESM.pdf]

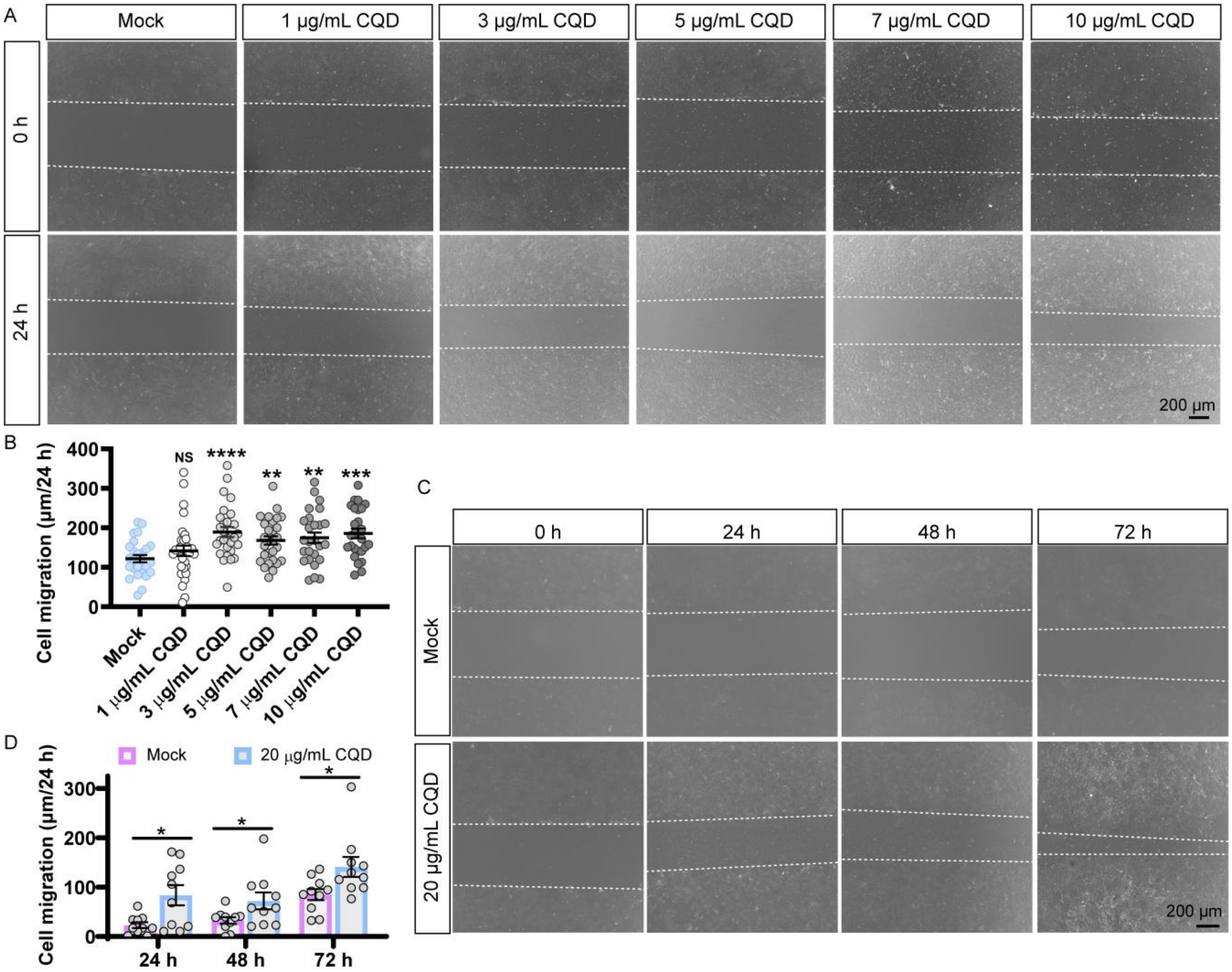

Fig. S2. CQD promotes cell migration. (A) Representative images illustrating the migration of Huh7.5.1 cells treated with concentrations of CQD ranging from 0 to 10 μg/mL for 24 hours. Scale bar: 200 μm. (B) Statistical analysis of cell migration distance, presented as means ± SEM from six independent experiments. (C) Representative images illustrating the migration of Huh7.5.1 cells treated with concentrations of 20 μg/mL CQD for 0 to 72 hours. Scale bar: 200 μm. (D) Statistical analysis of cell migration distance, presented as means ± SEM from six independent experiments.All data are reported as means ± SEM. Statistical significance was assessed using a two-tailed unpaired t-test, where NS indicates $p > 0.05$; * $p < 0.05$; ** $p < 0.01$; *** $p < 0.001$; **** $p < 0.0001$.
