# Supplementary material for: Carbon-based quantum dots enhance platelets aggregation through migrasomes biogenesis
Source: J Nanobiotechnology. 2026 Jan 17;24:152. doi: 10.1186/s12951-025-04010-9 (PMC12896335; doi:10.1186/s12951-025-04010-9)
Supplement: Supplementary file 3 — Supplementary Material 3 [file 12951_2025_4010_MOESM3_ESM.pdf]

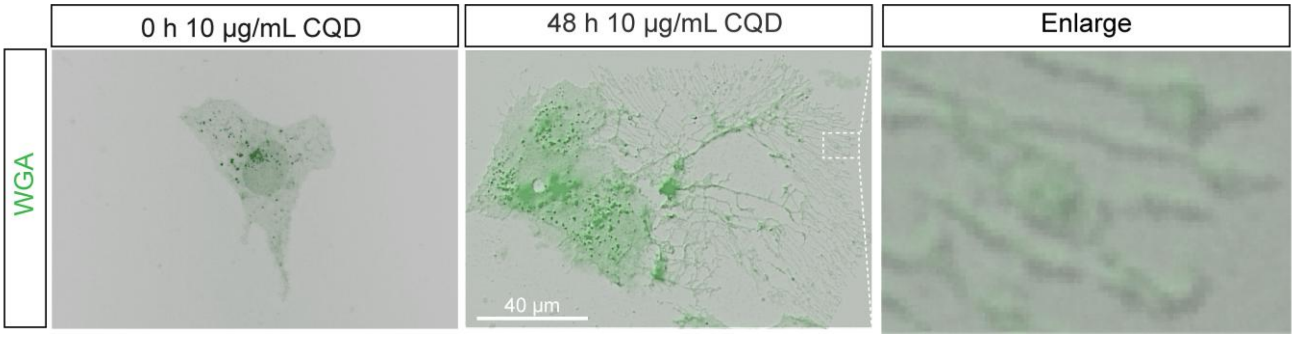

Fig. S3. CQD induces migrasome formation. Images depicting Huh7.5.1 cells stained with WGA after a 48-hour treatment with 10 μg/mL CQD. The representative images were translucently and merged with converted gray-value mode. Scale bar: 40 μm.
