# Supplementary material for: Carbon-based quantum dots enhance platelets aggregation through migrasomes biogenesis
Source: J Nanobiotechnology. 2026 Jan 17;24:152. doi: 10.1186/s12951-025-04010-9 (PMC12896335; doi:10.1186/s12951-025-04010-9)
Supplement: Supplementary file 4 — Supplementary Material 4 [file 12951_2025_4010_MOESM4_ESM.pdf]

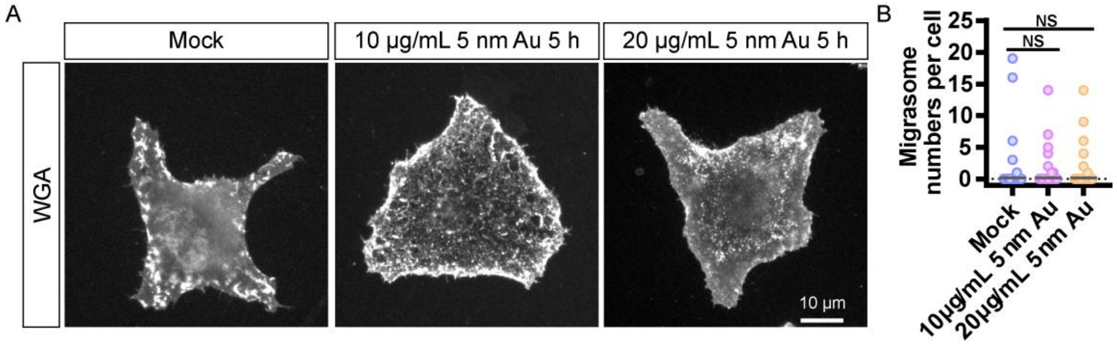

Fig. S4. Au nanoparticles do not increase migrasome formation. (A) Huh7.5.1 cells treated with 10 μg/mL and 20 μg/mL of 5 nm Au nanoparticles or a mock treatment for 5 hours were stained with WGA. Scale bar: 10 μm. (B) Statistical analyses of migrasome numbers per cell from penel A. NS denotes $p > 0.05$.
