# Supplementary material for: Carbon-based quantum dots enhance platelets aggregation through migrasomes biogenesis
Source: J Nanobiotechnology. 2026 Jan 17;24:152. doi: 10.1186/s12951-025-04010-9 (PMC12896335; doi:10.1186/s12951-025-04010-9)
Supplement: Supplementary file 5 — Supplementary Material 5 [file 12951_2025_4010_MOESM5_ESM.pdf]

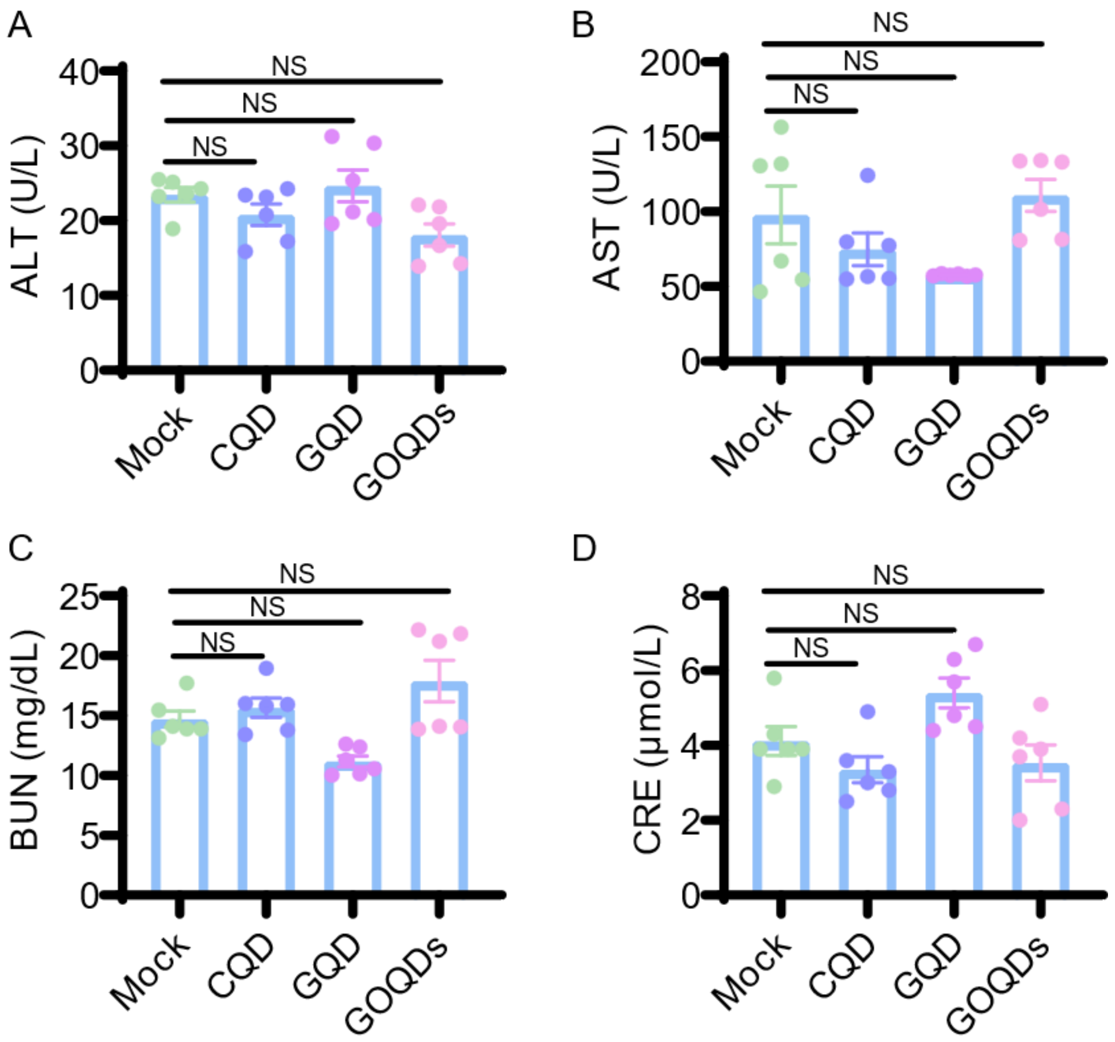

Fig. S5. Effects of CQD, GQD, and GOQDs on mouse liver and kidney function. (A) ALT levels in BALB/C mice after treatment with 3 μg/g body weight of CQD, 6 μg/g body weight of GQD, and 6 μg/g body weight of GOQDs for 7 days. (B) AST levels following treatment with 3 μg/g body weight of CQD, 6 μg/g body weight of GQD, and 6 μg/g body weight of GOQDs for 7 days. (C) BUN levels after treatment with CQD, GQD, and GOQDs for 7 days. (D) CRE levels after treatment with CQD, GQD, and GOQDs for 7 days.
